# GC-MS chemical profiling, antioxidant, anti-diabetic, and anti-inflammatory activities of ethyl acetate fraction of *Spilanthes filicaulis* (Schumach. and Thonn.) C.D. Adams leaves: experimental and computational studies

**DOI:** 10.3389/fphar.2023.1235810

**Published:** 2023-07-20

**Authors:** Oluwafemi Adeleke Ojo, Akingbolabo Daniel Ogunlakin, Gideon Ampoma Gyebi, Damilare IyinKristi Ayokunle, Adeshina Isaiah Odugbemi, Dare Ezekiel Babatunde, Omolola Adenike Ajayi-Odoko, Matthew Iyobhebhe, Samson Chukwuemeka Ezea, Christopher Oloruntoba Akintayo, Ademola Ayeleso, Adebola Busola Ojo, Omolara Olajumoke Ojo

**Affiliations:** ^1^ Phytomedicine, Molecular Toxicology, and Computational Biochemistry Research Laboratory (PMTCB-RL), Department of Biochemistry, Bowen University, Iwo, Nigeria; ^2^ Department of Biochemistry, Bingham University, Karu, Nigeria; ^3^ Department of Pure and Applied Biology, Bowen University, Iwo, Nigeria; ^4^ Department of Anatomy, Bowen University, Iwo, Nigeria; ^5^ Department of Microbiology, Bowen University, Iwo, Nigeria; ^6^ Department of Biochemistry, Landmark University, Omu-Aran, Nigeria; ^7^ Department of Pharmacognosy and Environmental Medicine, University of Nigeria, Nsukka, Nigeria; ^8^ Department of Physiology, Afe Babalola University, Ado-Ekiti, Nigeria; ^9^ Department of Life and Consumer Sciences, School of Agriculture and Life Sciences, University of South Africa, Roodepoort, South Africa; ^10^ Department of Biochemistry, Ekiti State University, Ado-Ekiti, Nigeria

**Keywords:** *Spilanthes filicaulis*, antioxidant, antidiabetic, anti-inflammatory, GC-MS profiling, molecular docking and dynamic simulations

## Abstract

**Introduction:** This study aimed to investigate the chemical profile of GC-MS, antioxidant, anti-diabetic, and anti-inflammatory activities of the ethyl acetate fraction of *Spilanthes filicaulis* leaves (EFSFL) via experimental and computational studies.

**Methods:** After inducing oxidative damage with FeSO_4_, we treated the tissues with different concentrations of EFSFL. An *in-vitro* analysis of EFSFL was carried out to determine its potential for antioxidant, anti-diabetic, and anti-inflammatory activities. We also measured the levels of CAT, SOD, GSH, and MDA.

**Results and discussion:** EFSFL exhibited anti-inflammatory properties through membrane stabilizing properties (IC_50_ = 572.79 μg/ml), proteinase inhibition (IC_50_ = 319.90 μg/ml), and inhibition of protein denaturation (IC_50_ = 409.88 μg/ml). Furthermore, EFSFL inhibited α-amylase (IC_50_ = 169.77 μg/ml), α-glucosidase (IC_50_ = 293.12 μg/ml) and DPP-IV (IC_50_ = 380.94 μg/ml) activities, respectively. Our results indicated that induction of tissue damage reduced the levels of GSH, SOD, and CAT activities, and increased MDA levels. However, EFSFL treatment restores these levels to near normal. GC-MS profiling shows that EFSFL contains 13 compounds, with piperine being the most abundant. *In silico* interaction of the phytoconstituents using molecular and ensembled-based docking revealed strong binding tendencies of two hit compounds to DPP IV (alpha-caryophyllene and piperine with a binding affinity of −7.8 and −7.8 Kcal/mol), α-glucosidase (alpha-caryophyllene and piperine with a binding affinity of −9.6 and −8.9 Kcal/mol), and to α-amylase (piperine and Benzocycloheptano[2,3,4-I,j]isoquinoline, 4,5,6,6a-tetrahydro-1,9-dihydroxy-2,10-dimethoxy-5-methyl with a binding affinity of −7.8 and −7.9 Kcal/mol), respectively. These compounds also presented druggable properties with favorable ADMET. Conclusively, the antioxidant, antidiabetic, and anti-inflammatory activities of EFSFL could be due to the presence of secondary metabolites.

## 1 Introduction

Researchers have explored various plants based on World Health Organization (WHO) authorization (World Health Organization, 1999). Researchers are using plants and their extracts to treat disorders such as diabetes and inflammation-related diseases (Andlib et al., 2023). Diabetes-related conditions rank among the top ten killer diseases in the world (Rendra et al., 2019). The symptoms of diabetes mellitus (DM) are caused by cellular dysfunction resulting from an imbalance between the antioxidant defense system and oxidative stress (Rendra et al., 2019). This reactive oxygen species (ROS) bonds with available proteins in a reactive process known as glycation. Diabetes is an elevation of blood glucose concentrations in the body (Cole and Florez, 2020). It is a form of the disease, that is, known to manifest when there is a perpetual and continuous increase in concentration, and it manifests in three major ways: first, this condition occurs when the pancreas is dysfunctional or even destroyed, and this state of condition is referred to as an “insulin-dependent condition” (Bellary et al., 2021). Because the beta cells present in the islets of Langerhans in the pancreas produce insulin. Second, this condition is also manifested when the body’s system is not responding to the insulin produced by the pancreas or when not enough insulin is produced to combat the increased concentration of glucose in the system (McIntyre et al., 2019; Bellary et al., 2021). This state of the disease has been linked to an increase in fat deposits over time due to people who lack exercise and those who have decided to live a sedentary lifestyle. This disease state is called insulin resistance, and it is the most common type of this disease condition because it occurs from childhood to adulthood (Bellary et al., 2021). Third, gestational diabetes, which is frequent among women, manifests itself in pregnant women when their glucose concentration in the system increases during pregnancy (McIntyre et al., 2019). The causative agent of this condition is free radicals, which initiate different manifestations of this disease, such as cardiomyopathy, nephropathy, neuropathy, and retinopathy. This disease condition has received a lot of interest in recent times, and several therapeutic means have been adopted by researchers to deal with it, such as the manufacture of antidiabetic drugs, including metformin (Ojo et al., 2022). But over the years, the drugs produced have not been able to fully manage the complications that arise from oxidative stress. The focus has now been on the use of organic therapies such as plants (Ojo et al., 2022). Furthermore, it has been identified that plants possess certain bioactive compounds, making them a powerful force in combating various diseases, including diabetes. These compounds, also known as phytochemicals, are known to also increase defense mechanism functions in the system, such as catalase (CAT), superoxide dismutase (SOD), and reduced glutathione (GSH), as well as suppress malondialdehyde (MDA) activities (Truong and Jeong, 2021). In the tropical and subtropical parts of the world, including Africa, America, Borneo, India, Sri Lanka, and Asia, *Spilanthes filicaulis* is also known as Creeping Spot Flower or African Cress (Tiwari et al., 2011). It is an annual plant that creeps and has prostrate stems that root from the nodes. They used seeds for reproduction. Their leaves are oval and alternating. On a short, somewhat hairy petiole, they tightly affixed the blade to the stem. The inflorescence is made up of short axillary peduncles with ovoid flower heads. They have blooms with golden rays and discs (Akoachere et al., 2015). In Babungo, northern Cameroon, the entire S. filicaulis plant is used to treat malaria, gastritis, toothaches, and stomachaches (Simbo, 2010). Additionally, the whole plant is used to cure chest discomfort, dermatitis, guinea worms, stomach problems, headaches, coughing, and toothaches. Additionally, it is used topically as a local anesthetic and as an enema to treat side discomfort (Ndenecho, 2009; Elufioye et al., 2019). This study aims to determine the antioxidant, antidiabetic, and anti-inflammatory properties of the ethyl acetate fraction of *S. filicaulis* leaves via experimental and computational studies.

## 2 Materials and methods

### 2.1 Chemicals and reagents

In this study, analytical grade solvents and reagents were used. Pancreatic α-amylase and α-glucosidase were obtained from Central Research Lab. Ltd, Ilorin. All chemicals used were of analytical grade.

### 2.2 Plant material and preparation of ethyl acetate fractions of *Spilanthes filicaulis* leaf

We obtained the leaves of S. filicaulis from Bowen University’s farmlands and identified them with the herbarium number BUH035. We cleaned the leaves to remove dirt and dust particles and left them to air dry for 2 weeks. Once dry, we processed them into a powdery form. Then, 30 g of powder was dissolved with an appropriate amount of methanol and water, and the mixture was allowed to macerate for 72 h. The resulting extract (MESFL) was filtered, concentrated, and stored at −20°C for further analysis. Subsequently, 20 g of MESFL was fractionated exhaustively with ethyl acetate to obtain the ethyl acetate fraction (EFSFL). We concentrated the ethyl acetate fraction using a rotary evaporator and stored the concentrate for further analysis.

### 2.3 *In vitro* antioxidant

#### 2.3.1 2,2-Diphenyl-1-picrylhydrazyl (DPPH) scavenging ability

We followed the procedure outlined by Ojo et al. (2022) to evaluate the ability of the extracts to scavenged DPPH radicals. For the comparative analysis, ascorbic acid served as the reference standard. The percentage of DPPH inhibition was determined by employing the following formula:

$$\%DPPH=\frac{{Abs}_{control}-{Abs}_{sample}}{{Abs}_{control}}\;x\;100$$

#### 2.3.2 Ferric reducing antioxidant power (FRAP) potential

The standard procedures of (Ojo et al., 2022; Ahmad et al., 2023) with a slight modification were used to measure the ability of EFSFL to reduce ferric ions. We measured the absorbance at 593 nm and we reported the outcome as µmol Fe (II)/g of the powder’s dry weight using the FeSO4 standard curve for calculation.

### 2.4 *In vitro* antidiabetic properties

#### 2.4.1 α-amylase inhibitory potential

This study followed the standard protocol (Ahmad et al., 2023) to determine the α-amylase inhibitory potential of EFSFL. To begin, we made a fresh preparation of enzyme, comprising 5 units per milliliter, in pH 6.7 ice-cold PBS with a concentration of 20 mM and including 6.7 mM NaCl. Then, 250 µL of the enzyme was combined with inhibitors (acarbose or EFSFL) at varying concentrations (excluding a blank sample), and the mixture was incubated at 37°C for 20 min. We subsequently added a starch solution with a concentration of 0.5% (w/v), and the mixture was incubated for an additional 15 min at 37°C. Immediately after the DNS reagent was added, the mixture was mixed and placed in a water bath at 100°C for 10 min. Finally, the absorbance was read at 540 nm.

#### 2.4.2 α-glucosidase inhibitory potential

The effect of EFSFL on intestinal α-glucosidase activity was evaluated using a technique described by (Bouslamti et al., 2023), which quantified the glucose generated by sucrose breakdown. To perform the assay, 100 µl of sucrose (50 mM), 1000 µl of phosphate buffer (50 mM; pH = 7.5), and 100 µl of α-glycosidase enzyme solution were prepared as the test solution (10 I.U.). Control (distilled water), positive control (acarbose), or EFSFL were all added to this mixture at varying concentrations. We read the absorbance of the ultimate solution at 500 nm.

#### 2.4.3 Dipeptidyl peptidase-IV (DPP-IV) activity

We carried out the inhibition of DPP-IV activity in 96-well ELISA plates. Evogliptin was used as the standard inhibitor. In each well, 30 μl of Tris-HCl buffer solution, 100 μL of enzyme DPP-IV, 100 μl of various concentrations (15.625, 31.25, 62.5 125, 250, 500, and 1000 μg/ml) of the fraction or standard, and 50 μL gly-pro-pnitroanilide as the substrate, were added. After adding the mixture, they incubated it for 30 min at 37°C, and then the absorbance was read by microplate readers at 405 nm (Bharti et al., 2012).

We calculated %inhibition using the formula:

$$\%\;Inhibition=\frac{DPP-IV\;activity\;\left(with\;fraction\right)}{DPP-IV\;activity\;\left(without\;fraction\right)}\;X\;100$$

### 2.5 Anti-inflammatory *in vitro* assays

#### 2.5.1 Human red blood cell (HRBC; RBC) membrane stability test

We conducted the procedure for the HRBC membrane stabilization assay following established protocols (Hogan et al., 2023). In summary, 2 ml reaction mixtures were prepared by combining varying amounts of EFSFL or a standard diclofenac drug with a 10% suspension of red blood cells. We kept these mixtures for 30 min at a temperature of 56 °C. Afterward, they were subjected to centrifugation at a speed of 2500 revolutions per minute (rpm) for 5 min. The resulting liquid above the sediment, known as the supernatant, was read for its absorbance at a wavelength of 560 nm.

#### 2.5.2 Protein denaturation inhibition

We carried out this assay to measure the inhibition of protein denaturation by following the procedure described by Hogan et al. (2023). The test sample included different concentrations of EFSFL and/or standard diclofenac, with a 10 µl 1% solution of bovine serum albumin. This mixture was heated to a temperature of 55°C for 30 min and left to cool. The turbidity of the samples was measured at a wavelength of 660 nm, and the extent to which the protein denaturation was prevented was calculated as the percentage of inhibition.

#### 2.5.3 Proteinase inhibitory assay

The proteinase inhibitory assay was carried out using the approach outlined in (Hogan et al., 2023). The procedure involved incubating 1 mL of the extract with a reaction mixture containing 2 ml of Tris-HCl buffer and 0.06 mg trypsin at 37°C for 5 min. Then, 0.8% (w/v) casein was added to the reaction mixture and allowed to incubate for 20 min. The reaction was stopped by adding 2 ml of 70% perchloric acid, and the resulting supernatant was measured for absorbance at 210 nm after centrifugation.

### 2.6 *Ex-vivo* studies

#### 2.6.1 Rat experiments and organ harvesting

The study utilized healthy male Wistar rats, weighing between 200 and 250 g each, obtained from the Anatomy Department at Bowen University in Iwo, Nigeria. Before being put to death using ketamine, the rats underwent an overnight fasting period. The livers were then extracted and blended in a 50 mM phosphate buffer solution containing 1% Triton X-100. For *ex-vivo* research purposes, we collected the supernatants in plain tubes after centrifugation at 15,000 rpm and 40°C. The study was approved under a specific identification number (BUAC/BCH/2023/0001A), and the protocols approved by Bowen University’s institutional animal ethics committee were followed when caring for the rats.

#### 2.6.2 Induction of liver damage

The protocol defined by Ojo et al. (2022) was employed to induce liver injury *ex vivo* in experimental rats. To perform this, the organ supernatant, which had different concentrations of EFSFL (varying between 31.25 μg/ml to 1000 μg/mL), was mixed with 200 μL. We added 100 μL of a solution containing 0.1 mM FeSO_4_. We then placed the resulting mixture in a 37°C environment for 30 min to allow for biochemical analysis. In the normal control, only the organ supernatant was used in the reaction mixture, while the negative control comprises the tissue supernatant and FeSO_4_.

### 2.7 *Ex vivo* analysis

#### 2.7.1 Catalase (CAT) activity

We assessed the CAT activity assay of EFSFL with slight modifications to the method described by Ojo et al. (2022). Tissue samples containing different concentrations of EFSFL were mixed with 780 μl of 50 mM phosphate buffer, followed by the addition of 300 μl of 2 M H_2_O_2_. For 3 minutes, we read the absorbance at 240 nm at 1-min intervals.

#### 2.7.2 Superoxide dismutase

We used the method provided in (Ajiboye et al., 2019) to determine SOD activity. To summarize, we mixed 170 μl of diethylenetriaminepentaacetic acid and 15 μl of the incubated sample in a test tube. Then, we added 15 μl of 6-hydroxydopamine to the solution and gently shook it. Finally, we measured the solution at 492 nm for 3 min with a 1-min interval.

#### 2.7.3 Reduced glutathione level

Based on the protocol depicted in (Ajiboye et al., 2019), the tissue lysates, with a volume of 600 μl, were treated to remove proteins by adding 600 μl of a solution containing 10% TCA. After 10 min of centrifuging the mixture at 3500 rpm, 500 μl of the sample was transferred to a new test tube. Next, 100 μl of Ellman reagent was added to the sample, and the mixture was allowed to incubate at a temperature of 25°C for 5 min. We subsequently measured the absorbance of the solution at a wavelength of 415 nm.

#### 2.7.4 Lipid peroxidation level

The ability of EFSFL to inhibit lipid peroxidation by following the method outlined in reference (McIntyre et al., 2019) They carried out the process by taking 100 μl of tissue lysates that contained varying concentrations of EFSFL. We added 1000 μl of 0.25% thiobarbituric acid, 100 μl of 8.1% SDS, and 375 μl of 20% acetic acid to it. We boiled the mixture at 95°C for an hour in a water bath. After cooling it to room temperature, they measured its absorbance at 532 nm.

### 2.8 Molecular docking studies of GCMS identified compounds against α-amylase, dipeptidyl peptidase IV (DPP IV), and α-glucosidase

#### 2.8.1 Protein structure preparation

The retrieval of protein structures was from the Protein Data Bank (http://www.rcsb.org) for the deposited three-dimensional structures of human dipeptidyl peptidase IV (DPP IV) complexed with evogliptin (PDBID: 5Y7K), human pancreatic α-amylase (HPA) complexed with acarbose (PDBID: 1B2Y), and human α-glucosidase complexed with acarbose (HG) (PDB ID: 3TOP). The existing ligands and water molecules were removed from all the crystal structures while missing hydrogen atoms were added using MGL-AutoDock Tools (ADT, v1.5.6) (Morris et al., 2009).

#### 2.8.2 Ligand preparation

The retrieval of Structure Data Format (SDF) of acarbose (reference inhibitors) and 13 phytocompounds identified by GC-MS analyses of EFSFL were downloaded from the PubChem database (www.pubchem.ncbi.nlm.nih.gov) before their conversion to PDB chemical format using Open Babel (O'Boyle et al., 2011). Non-polar hydrogen molecules were merged with the carbons, while the polar hydrogen charges of the Gasteiger-type were assigned to atoms. Furthermore, ligand molecules were converted to dockable PDBQT format with the help of AutoDock Tools.

#### 2.8.3 Validation of molecular docking protocol

The virtual screening docking protocol was validated by aligning the docked poses of the native ligands (acarbose and evogliptin) with the extracted co-crystallized ligand from both proteins, which had the lowest binding affinity from the initial docking. We calculated the RMSD using Discovery Studio Visualizer, BIOVIA, 2020.

#### 2.8.4 Molecular docking of phytochemicals with the targeted active site

The active site targeted molecular docking with the reference inhibitors and the GC-MS identified compounds against DPP IV, HPA, and HG was performed using AutoDock Vina in PyRx 0.8 (Trott and Olson, 2010). For the docking analysis, the ligands were imported, and energy minimization was accomplished using Open Babel (O'Boyle et al., 2011) incorporated into PyRx 0.8. The Universal Force Field (UFF) and conjugate gradient descent were employed as the energy minimization parameter and optimization algorithm, respectively. Although other parameters were left at their default values, the binding site coordinates of the target enzymes are shown in Supplementary Table S1, and the molecular interactions were viewed using Discovery Studio Visualizer version 16.

#### 2.8.5 Molecular dynamics

For ensemble-based docking, the DPP IV, HPA, and HG apoenzymes were subjected to a 50 ns simulation of molecular dynamics. The MD trajectory obtained was also used in cluster analysis. GROMACS 2019.2 and GROMOS96 43a1 forcefield (Lee et al., 2016; Lee et al., 2017; Lee et al., 2020) were used for the analysis. We generated protein and ligand topology files using the Charmm GUI (Lee et al., 2016; Lee et al., 2020)**.** The solvation system, periodic boundary conditions, physiological conditions, minimization of the systems, and equilibration in a constant number of atoms, constant pressure, and constant temperature (NPT) used in the simulation are similar to those in our previous report (Gyebi et al., 2021; Ogunyemi et al., 2021; Ogunlakin et al., 2023; Ogunyemi et al., 2023). Velocity rescales and Parrinello-Rahman barostat were used to maintain the temperature and pressure at 310 K and 1 atm, respectively. We used a 2-femtosecond time step with a leap-frog integrator. Each system underwent a 100 ns simulation, with snapshots taken every 0.1 nanosecond and totaling 1000 frames for each system. From the MDs trajectories, the RMSD and RMSF were computed and presented as Supplementary Material.

#### 2.8.6 Molecular dynamic trajectory clustering of unattached proteins

A representative conformation that was obtained from the generated cluster from the clustering of the 500 ns MD trajectories of the unbound enzymes was employed for ensemble-docking studies. We performed the MD simulation trajectory clustering using TTClust V 4.9.0 (Lee et al., 2020). The systems were automatically clustered using the Python TTClust package, which uses the elbow approach to establish the ideal number of clusters before generating a representative frame for each cluster.

#### 2.8.7 Ensembled-based docking of the GCMS identified phytochemicals to different conformations of the enzymes

Using the AutoDock Vina program (Trott and Olson, 2010), the phytocompounds identified by GCMS were docked to the various representative conformers of DPP IV (4), HPA (2), and HG (4). The average binding affinities of the phytochemicals to each of the proteins were computed from the binding affinities of the phytochemicals to each of the conformations of the enzymes. The average binding energies of the compounds for the two targets were calculated and then their affinities were scored. The Discovery Studio Visualizer, BIOVIA 2020, was used to see how the lead chemicals interacted with one another on a molecular level.

#### 2.8.8 Physicochemical properties and ADMET in silico study

The top two phytochemicals of each protein were then analyzed for ADMET filtering and drug-likeness using a variety of descriptors. SwissADME was used for the drug-likeness analysis using Lipinski filtering methods (http://www.swissadme.ch/index.php) on the webserver, while the anticipated toxicity, distribution, metabolism, and absorption (ADME/tox) study was analyzed with the SuperPred webserver (http://lmmd.ecust.edu.cn/admetsar1/predict/). The SDF file and the canonical SMILES of the compounds were downloaded from the PubChem database or copied from ChemDraw to calculate the ADMET properties using default parameters.

### 2.9 Statistical analysis

The data was analyzed with software called GraphPad Prism version 9.0.1. We reported descriptive statistics as mean ± SD. GraphPad was used to analyze the results, which are presented as graphs. To compare the means, a statistical method known as one-way ANOVA was followed by Tukey’s *post hoc* test with a significance level of *p* < 0.05.

## 3 Results

### 3.1 1,1-Diphenyl-2-picrylhydrazyl (DPPH) quenching ability

**FIGURE 1:**
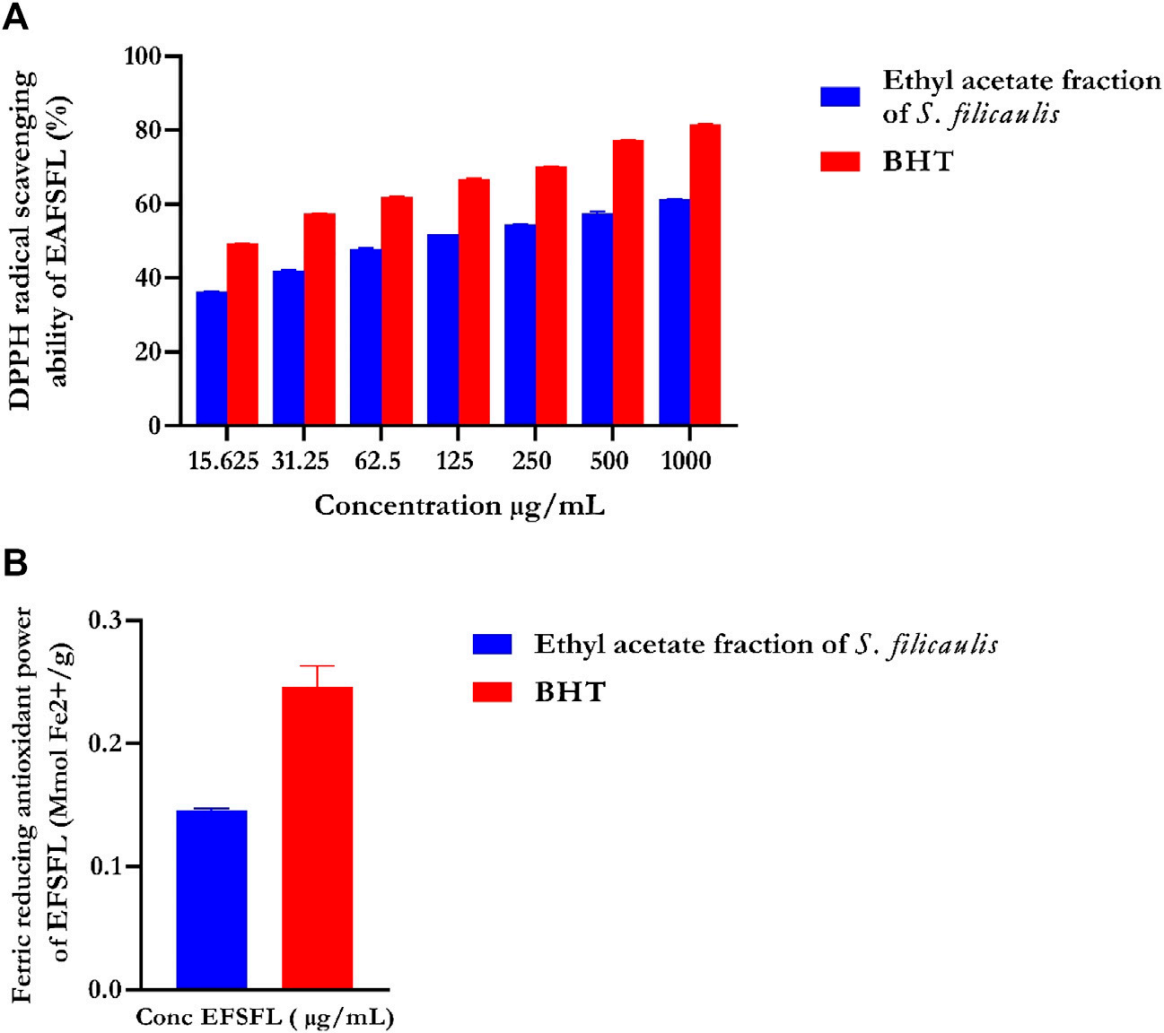
*In vitro* antioxidant activities of ethyl acetate fraction of *S. filicaulis* leaf. **(A)** DPPH radical scavenging ability and **(B)** ferric reducing antioxidant power. Legends: Data are represented as mean ± SD (n = 3). EFSFL: ethyl acetate fraction of *S. filicaulis*; BHT: butylated hydroxytoluene.

DPPH, an unvarying radical, is commonly utilized to assess the effectiveness of antioxidants derived from plant sources. Figure 1A illustrates the percentage inhibition of DPPH scavenging ability by EFSFL at different concentrations. EFSFL exhibited a significant ability to counteract the DPPH radical, with an IC50 value of 46.40 ± 5.31 μg/mL. Comparatively, the IC_50_ value of butylated hydroxytoluene (BHT), was 15.76 ± 0.58 μg/ml, as indicated in Table 1.

**TABLE 1:** IC_50_ values of ethyl acetate fraction of *S. filicaulis* leaves against DPPH, FRAP, α-amylase, α-glucosidase, membrane stabilization, protein denaturation, and proteinase inhibition.

Activity	Plant extract/Standard	IC_50_ (µg/ml)
DPPH	EFSFL	274.32
	BHT	16.11
α-amylase	EFSFL	169.77
	Acarbose	121.79
α-glucosidase	EFSFL	293.12
	Acarbose	189.75
Dipeptidyl peptidase-IV	EFSFL	380.94
	Evogliptin	211.35
Membrane stabilization	EFSFL	572.79
	Diclofenac	3.74
Protein denaturation	EFSFL	409.88
	Diclofenac	58.90
Proteinase inhibition	EFSFL	319.90
	Diclofenac	154.66

EFSFL, ethyl acetate fraction of *S. filicaulis*

### 3.2 Ferric reducing antioxidant power (FRAP) of EFSFL

The FRAP assay was used to assess the ferric-reducing potential of EFSFL. The results demonstrated increased FRAP activities at the highest concentration studied, 1000 μg/ml. Standard butylated hydroxytoluene (BHT) showed the highest reducing property compared to MESFL (Figure 1B).

### 3.3 *In vitro* anti-diabetic studies

#### 3.3.1 Evaluation of α-amylase inhibition

**FIGURE 2:**
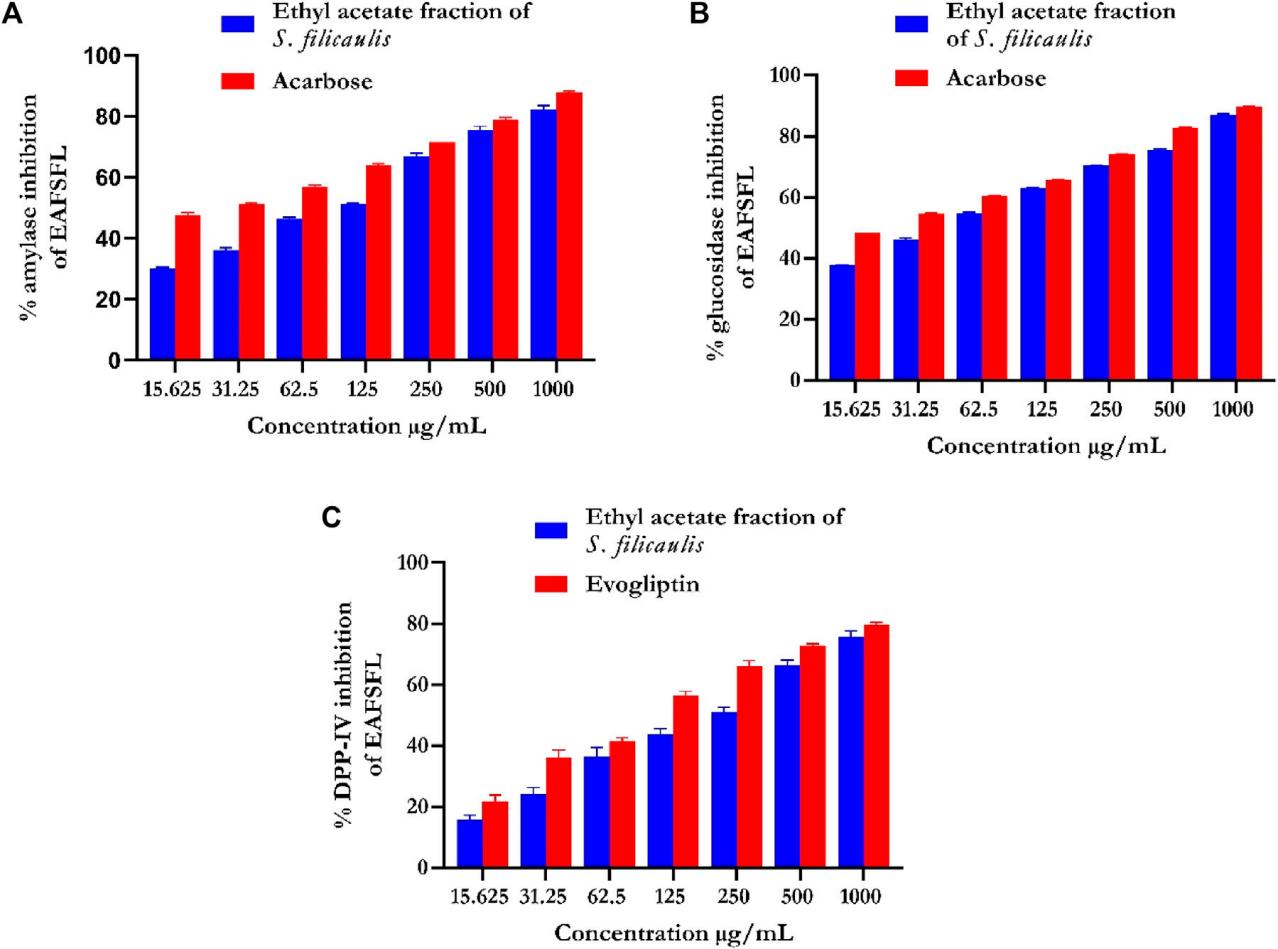
*In vitro* antidiabetic activities of ethyl acetate fraction of *S. filicaulis* leaf. **(A)** α-amylase inhibition, **(B)** α-glucosidase inhibition, and **(C)** dipeptidyl peptidase-4 (DPP-4) inhibition. The bar chart illustrates the percentage of proteinase activity inhibition. The data from three separate assays are presented as mean ± SD.

EFSFL efficiently blocked α-amylase in a concentration-dependent manner and had an IC_50_ value of 307.02 ± 4.25 μg/ml compared with those of the standard (Figure 2A; Table 1). Acarbose revealed an 87.94% inhibitory property of α-amylase (IC_50_ 121.79 ± 2.26 μg/mL) (Figure 2A; Table 1).

#### 3.3.2 Evaluation of α-glucosidase inhibition

EFSFL efficiently inhibited α-glucosidase at all concentrations tested and an IC_50_ value of 215.51 ± 0.47 μg/ml (Figure 2B; Table 1). Acarbose revealed an 89.68% inhibitory property of α-glucosidase (IC_50_ 189.79 ± 0.67 μg/ml) (Figure 2B; Table 1).

#### 3.3.3 Evaluation of DPP-IV inhibitory activity of ethyl acetate fraction of S. filicaulis leaf

Figure 2C shows the DPP-IV inhibitory activity of EFSFL. With an IC_50_ value of 380.94 μg/mL (Table 1), EFSFL inhibited DPP-IV in a concentration-dependent manner. The EFSFL DPP-IV inhibitory activity, however, was less potent than that of the standard DPP-IV inhibitor, evogliptin (IC_50_ = 211.35 μg/ml).

### 3.4 *In vitro* anti-inflammatory analysis

#### 3.4.1 Human red blood cell (RBC; HRBC) membrane stabilization test

**FIGURE 3:**
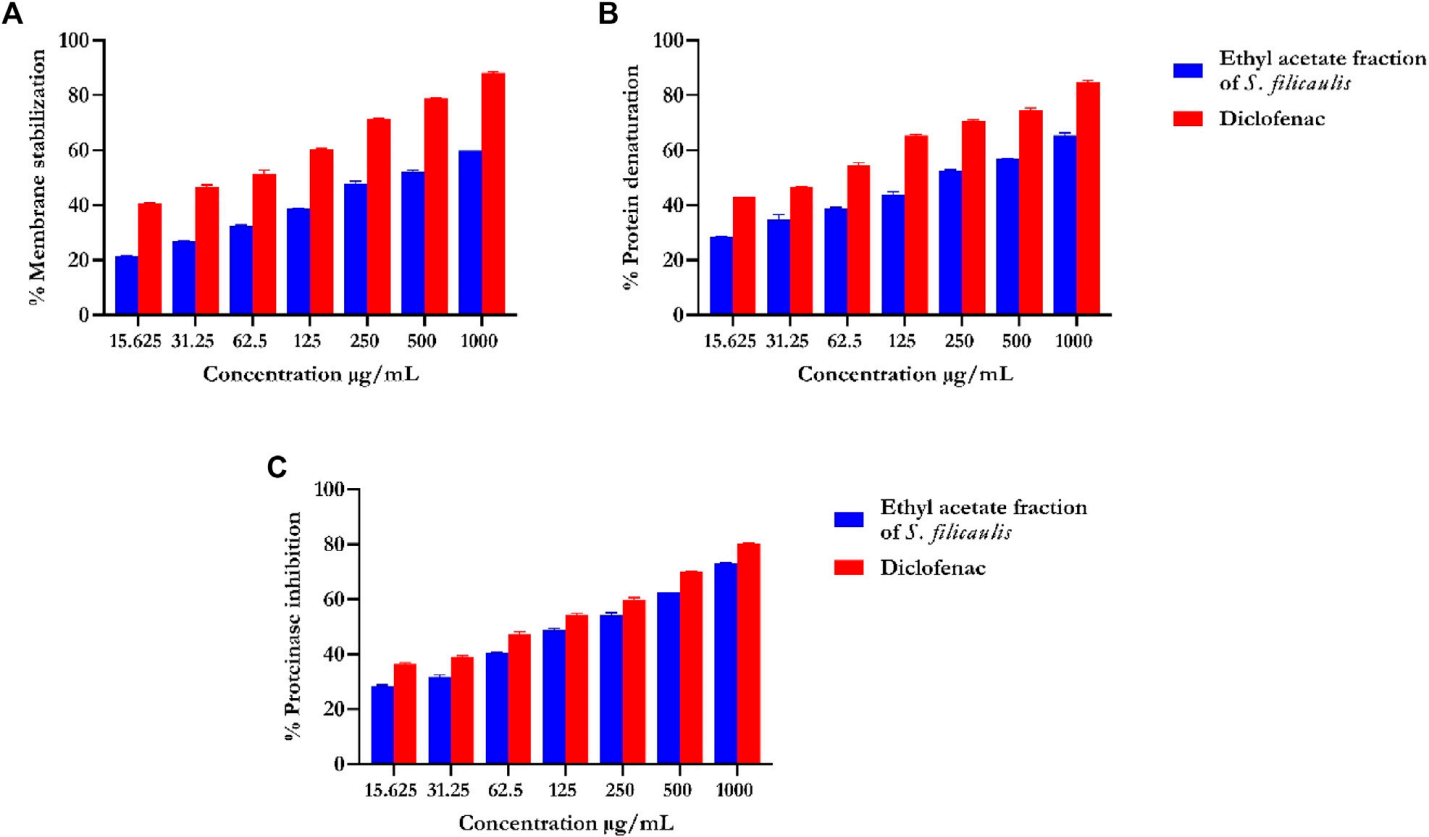
*In vitro* inflammatory activities of ethyl acetate fraction of *S. filicaulis* leaf. **(A)** Membrane stabilization, **(B)** inhibition of protein denaturation, and **(C)** proteinase inhibitory activity by Legend: The bar chart illustrates the percentage of proteinase activity inhibition. The data from three separate assays are presented as mean ± SD (n = 3).

Our data revealed that the EFSFL demonstrated HRBC membrane stabilization potential (IC_50_ = 319.85 ± 4.54 μg/mL) in a concentration-dependent manner. The standard drug, diclofenac, showed 89.68% membrane stabilization potential (IC_50_ 3.63 ± 1.32 μg/mL) (Figure 3A; Table 1).

#### 3.4.2 Inhibition of protein denaturation

Our analysis also revealed concentration-dependent increases in the inhibition of protein denaturation (IC_50_ = 72.75 ± 11.06 μg/mL) by EFSFL. The standard drug, diclofenac, also exhibited protein denaturation inhibition (IC_50_ = 59.13 ± 12.40 μg/mL) (Figure 3B; Table 1).

#### 3.4.3 Proteinase inhibitory assay

Our analysis revealed that EFSFL exhibited significant proteinase inhibitory activities ((IC_50_ = 296.08 ± 11.47 μg/mL). The standard drug, diclofenac, also exhibited proteinase inhibitory activities (IC_50_ = 154.62 ± 4.29 μg/mL) (Figure 3C; Table 1).

### 3.5 *Ex vitro* antioxidant analysis

#### 3.5.1 Evaluation of catalase activity

**FIGURE 4:**
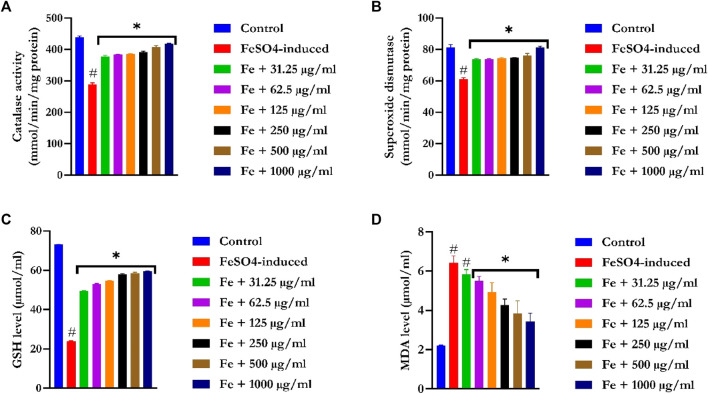
*Ex vivo* antioxidant activities of ethyl acetate fraction of *S. filicaulis* leaf in Fe^2+^-induced liver damage. **(A)** Catalase activity, **(B)** superoxide dismutase activity, **(C)** GSH level, and **(D)** MDA level. Legend: Data are represented as mean ± SD (n = 3).

Figure 4A showed a significant (*p* < 0.05) reduction in CAT activities in the liver tissues of FeSO_4_-induced animals. Treatment with EFSFL concentration-dependently (*p* < 0.05) increased the activity in a significant manner, with the most pronounced activity found in the 1000 μg/ml treated group.

#### 3.5.2 Evaluation of superoxide dismutase activity

SOD activities were significantly (*p* < 0.05) reduced in the liver tissues of FeSO_4_-induced animals (Figure 4B). Treatment with EFSFL dose-dependently (*p* < 0.05) elevated SOD activity in a manner close to that of the normal group.

#### 3.5.3 Evaluation of reduced glutathione

GSH levels were drastically (*p* < 0.05) decreased in the liver tissues of FeSO_4_-induced animals (Figure 4C). In contrast, EFSFL-treated groups significantly (*p* < 0.05) increased the levels of GSH in a manner close to the normal group, with the most pronounced effect found in the 1000 μg/ml treated group.

#### 3.5.4 Determination of malondialdehyde level

The MDA level was notably increased in the liver tissues of FeSO_4_-induced animals (Figure 4D). In contrast, EFSFL-treated groups significantly (*p* < 0.05) reduced the level of malondialdehyde to near normal, with the most striking effect found in the 1000 μg/ml treated group.

### 3.6 GC-MS analysis

EFSFL underwent GC-MS analysis to determine its phytoconstituents. By contrasting the GC-MS spectra with a reference library (NIST), 13 compounds were identified and are listed in Table 2. The two most abundant compounds in EFSFL were piperine (16.45%) and ocimene (10.73%). Cycloheptasiloxane, tetradecamethyl- (3.96%) oleic acid (3.96%0, and 2-Methyl-Z,Z-3,13-octadecadienol (3.72%) were found in small amounts, whereas Benzocycloheptano[2,3,4-I,j]isoquinoline, 4,5,6,6a-tetrahydro-1,9-di hydroxy-2,10-dimethoxy-5-methyl- (−0.09%), erucic acid (0.82%), spilanthol (0.80%), octadec-9-enoic acid (0.38%), petroselaidic acid (0.17%), α-caryophyllene (0.58%), 2-methyl-Z,Z-3,13-octadecadienol (0.89%), and cis-9-Hexadecenoic acid (0.57%) were present in minute amounts. The GC-MS chromatograms of the phytocompounds are displayed in the Supplementary Material (Supplementary Material S1).

**TABLE 2:** GC-MS predicted compounds of ethyl acetate fraction of *S. filicaulis* leaves with their molecular weight, formula, and peak area (%).

S/N	R.T	Compound	Molecular formula	Molecular weight	Area (%)
1	11.142	Cycloheptasiloxane, tetradecamethyl-	C_14_H_42_O_7_Si_7_	519.08	3.96
2	12.247	Benzocycloheptano[2,3,4-I,j]isoquinoline, 4,5,6,6a-tetrahydro-1,9-di	C_20_H_23_NO_4_	314.40	0.09
		hydroxy-2,10-dimethoxy-5-methyl-			
3	12.472	Erucic acid	C_22_H_42_O_2_	338.60	0.82
4	12.994	Spilanthol	C_14_H_23_NO	221.34	0.80
5	13.159	Octadec-9-enoic acid	C_18_H_34_O_2_	282.50	0.38
6	13.243	Petroselaidic acid	C_18_H_34_O_2_	282.50	0.17
7	13.486	α-caryophyllene	C_15_H_24_	204.35	0.58
8	14.071	Oleic Acid	C_18_H_34_O_2_	282.50	3.96
9	14.680	2-Methyl-Z,Z-3,13-octadecadienol	C_19_H_36_O	280.50	3.72
10	15.098	cis-9-Hexadecenoic acid	C_16_H_30_O_2_	254.41	0.89
11	15.312	Pinene	C_10_H_16_	136.23	0.57
12	16.776	Piperine	C_17_H_19_NO_3_	284.34	16.45
13	17.078	Ocimene	C_10_H_16_	136.23	10.73

### 3.7 Molecular docking studies

#### 3.7.1 Docking protocol validation

The protocol to be used was validated to predict the precision, reliability, and accuracy of the docking protocol (Ogunyemi et al., 2023) before the docking of the GCMS-identified compounds to the target proteins. The generated docked poses of the reference compounds having the least energetic conformation were superimposed on the native ligand that was co-crystallized. After the superimposition, the root-mean-square deviation (RMSD) was computed. The RMSD, or acarbose complexed with 5y7K and 3top, was 3.5651 and 0.4341 Å respectively. The low RMSD shows that the docking protocol was suitable for the docking of phytochemicals (Supplementary Figure S2).

#### 3.7.2 Molecular docking of identified compounds against human dipeptidyl peptidase IV, α-amylase, and α-glucosidase

Table 3 shows the docking affinities of the 13 GCMS-identified phytochemicals from EFSFL and the reference molecule (acarbose) against DPP IV, HPA, and HG. Based on the lowest binding energies, binding poses, and interactions in the catalytic site, the top two ranked phytocompounds for each enzyme were selected for interactive analysis (Table 4). The two top docked phytocompounds to the DPP IV are alpha-caryophyllene and piperine, with binding affinity values of −7.8 and −7.8 Kcal/mol, respectively, compared to the reference inhibitor (evogliptin) (−7.8 Kcal/mol). Alpha-caryophyllene and piperine were the top-ranked phytocompounds for HG, with binding affinities of −9.6 and −8.9 Kcal/mol, respectively, compared to the reference inhibitor (acarbose), which had a binding affinity of 10.6 Kcal/mol. For HPA, the top-ranked phytocompounds were piperine and benzocycloheptano[2,3,4-I,j]isoquinoline, 4,5,6,6a-tetrahydro-1,9-dihydroxy-2,10-dimethoxy-5-methyl, with binding affinities of −7.8 and −7.9 Kcal/mol, compared to acarbose, which had a binding affinity of −8.3 Kcal/mol. The results from the initial docking studies showed that alpha-caryophyllene and piperine had high multi-target binding tendencies (Table 4).

**TABLE 3:** Binding energies of GCMS phytoconstituents identified from ethyl acetate fraction of *S. filicaulis* leaves against human dipeptidyl peptidase IV (DPP IV), α-amylase and α-glucosidase.

S/No	Compound	Binding energy (Kcal/mol)		
		DPP IV	HG	HPA
S1	Acarbose_1b2y (E = 376.73)		−10.6	−8.3
S2	Evogliptin (E = 415.60)	−7.8		
1	alpha-caryophyllene (E = 1912.23)	−7.8	−9.6	−7.4
2	Piperine (E = 356.64)	−7.8	−8.9	−7.8
3	Petroselaidic_acid (E = 84.42)	−7.5	−8.8	−5.3
4	Pinene (E = 661.52)	−5.8	−6.6	−5.5
5	2-Methyl-Z,3, 13-octadecadienol (E = 123.57)	−4.9	−6.4	−5.9
6	Octadec-9-enoic_acid (E = 69.17)	−4.7	−6.2	−5.6
7	Spilanthol (E = 103.48)	−4.9	−6.1	−6
8	Erucic_acid (E = 99.56)	−5.7	−6.1	−5.4
9	Cycloheptasiloxane, tetradecamethyl	−5.6	−6.1	−5.2
10	Ocimene (E = 113.15)	−5	−6.1	−5.1
11	Benzocycloheptano[2,3,4-I,j]isoquinoline, 4,5,6,6a-tetrahydro-1,9-di hydroxy-2,10-dimethoxy-5-methyl (*BCQDM*)	−5.7	−6	−7.9
12	cis-9-Hexadecenoic_acid (E = 85.30)	−5.2	−5.9	−5.6
13	Oleic_Acid (E = 81.73)	−4.5	−5.7	−5.5

**TABLE 4:** Top two ranked compounds from the molecular docking of the GCMS-identified phytoconstituents from ethyl acetate fractions of *S. filicaulis* leaves against human dipeptidyl peptidase IV (DPP IV), α-amylase and α-glucosidase.

S/No	Name	Structure
	Acarbose	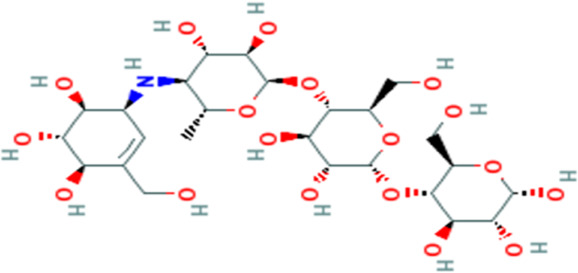
1	Evogliptin	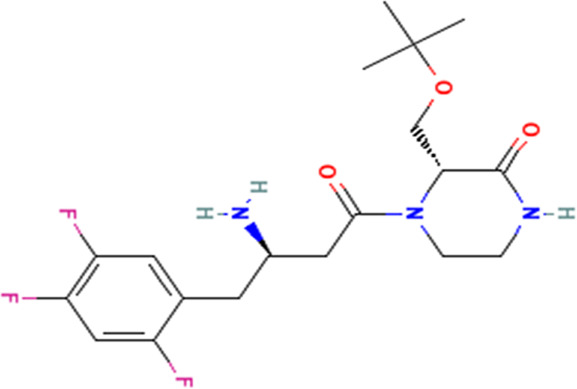
1	Piperine	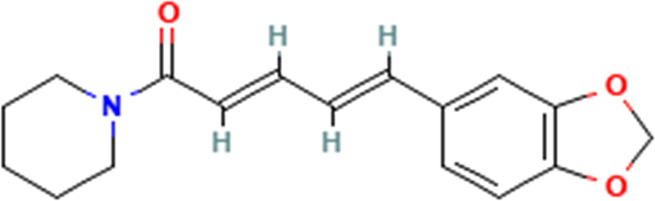
2	Benzocycloheptano[2,3,4-I,j]isoquinoline,_4,5,6,6a-tetrahydro-1,9-dihydroxy-2,10-dimethoxy-5-methyl (*BCQDM*)	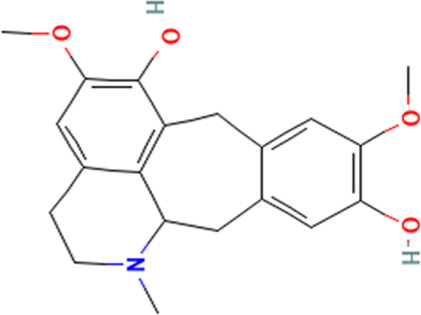
3	α-caryophyllene	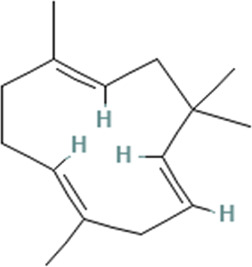

#### 3.7.3 Amino acid interaction of top docked compounds with human dipeptidyl peptidase IV (DPP IV), α-amylase, and α-glucosidase

**FIGURE 5:**
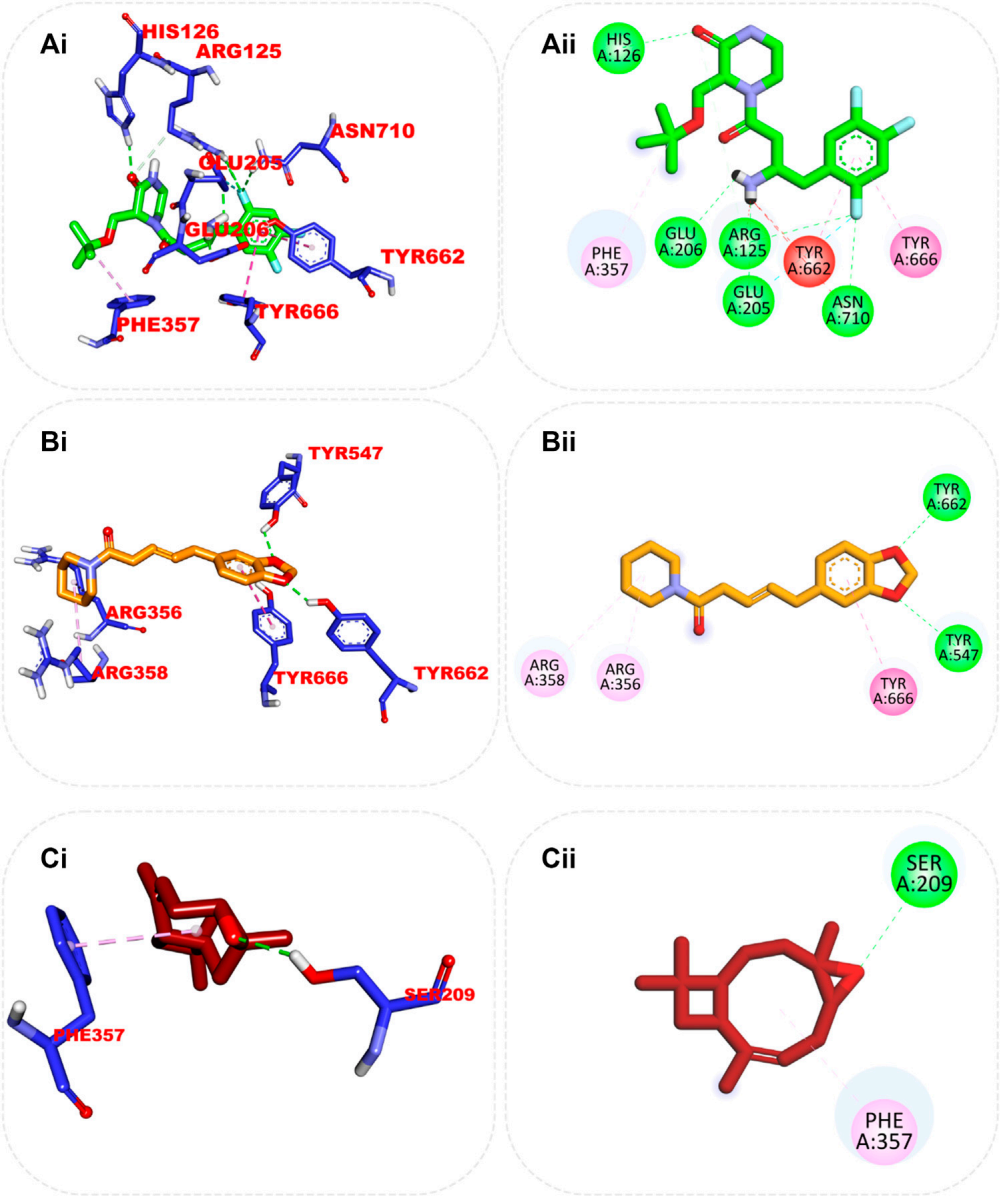
Top ranked secondary metabolites and reference blocker (acarbose) from the docking analysis of GCMS phytochemicals from ethyl acetate fraction of *S. filicaulis* leaves in a human’s active site dipeptidyl peptidase IV. The ligands are displayed as sticks and distinguished by their colors **(Ai)** 3D interaction of evogliptin (reference inhibitor) is presented in green, **(Aii)** 2D interaction of evogliptin, **(Bi)** 3D interaction of alpha-caryophyllene is presented in gold **(Bii)** 2D interaction of alpha-caryophyllene, and **(Ci)** 3D interaction of piperine is shown in red **(Cii)** 2D interaction of piperine.

The interaction of the reference compound and two top-docked phytochemicals with amino acids of the catalytic residues of DPP IV, HPA, and HG is represented in Table 5. The interaction of respective ligand groups with residues of the enzymes was majorly hydrophobic, with a few H-bonds below (less than 3.40 Å). Top-docked compounds were oriented in the active site of DPP IV and interacted with the amino acid to which we docked the reference compound. The interaction between alpha-caryophyllene and DPP IV was stabilized by a hydrogen bond with Ser209 and a hydrophobic contact with Phe357, while piperine formed two hydrogen bonds with Tyr662 and Tyr547 and pi-alkyl hydrophobic Tyr666, Arg358, and Arg356 (Figure 5).

**TABLE 5:** Interaction of amino acid residues of dipeptidyl peptidase IV, α-amylase, and α-glucosidase with the top two GCMS-identified phytoconstituents from ethyl acetate fraction of *S. filicaulis* leaves.

Compounds	Protein		Hydrogen bonds (Bond distance (Å))		Hydrophobic interaction (Bond distance (Å))
		Numbers	Interacting residues	Numbers	Interacting residues
Evogliptin	DPP IV	8	His126 Glu206 Arg125 Glu205 Ans10	3	Phe357 Tyr666 Tyr662
alpha-caryophyllene		1	Ser209	1	Phe357
Piperine		2	Tyr662 Tyr547	3	Tyr666 Arg358 Arg356
Acarbose	HPA	18	Trp59 (2) Gln63 Tyr62 Thr163 Arg195 Asp197 Lys200 (2) Glu233 Asp300 Gly30 His299 Glu233 Ile235 (2) Glu240 Gly306 His305	1	Trp59
Piperine		0		3	Tyr62 Gln63 Trp59 Ala198
Benzocycloheptano[2,3,4-I,j]isoquinoline, 4,5,6,6a-tetrahydro-1,9-di			Asp197 Glu233 Arg195 Ala198 His305	2	Asp300 Tyr62
hydroxy-2,10-dimethoxy-5-methyl (BCQDM)					
Acarbose	HG	14	Arg1582 Arg1510 Asp1526 Tyr1167 Met1421 Asp1157 Lys1460 Gln1561 Thr1528 Trp1355 Asp1279 His1584 Asp1317 Asp1555	3	Tyr1251 Phe1559 Phe1560
Piperine		2	Lys1460 Arg1510	5	Phe1427 Trp1369 Ile1280 Trp1355 Tyr1251
alpha-caryophyllene		0		2	Phe1560 Trp1355

**FIGURE 6:**
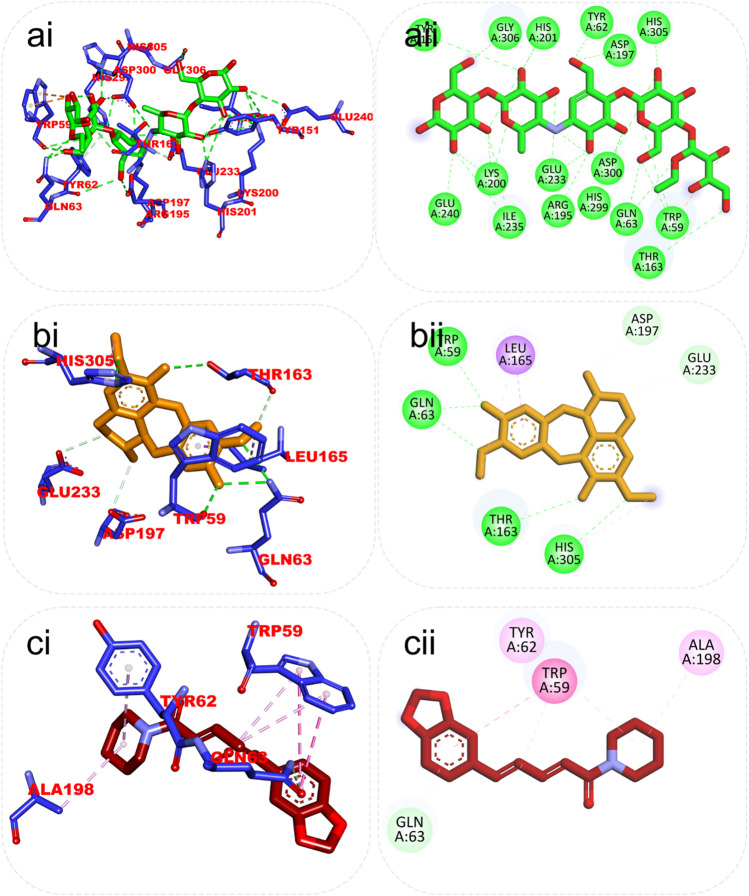
Top ranked secondary metabolites and reference inhibitor (acarbose) from the docking analysis of GCMS phytochemicals from ethyl acetate fraction of *S. filicaulis* leaves found to interact with the active site of *human* α-amylase. The ligands are displayed as sticks and distinguished by their colors **(Ai)** 3D interaction of acarbose is shown in green, **(Aii)** 2D interaction of acarbose, **(Bi)** 3D interaction of Benzocycloheptano[2,3,4-I,j]isoquinoline, 4,5,6,6a-tetrahydro-1,9-dihydroxy-2,10-dimethoxy-5-methyl is shown in gold, **(Bii)** 2D interaction of Benzocycloheptano[2,3,4-I,j]isoquinoline, 4,5,6,6a-tetrahydro-1,9-dihydroxy-2,10-dimethoxy-5-methyl, and **(Ci)** 3D interaction of piperine is shown in red, **(Cii)** 2D interaction of piperine.

**FIGURE 7:**
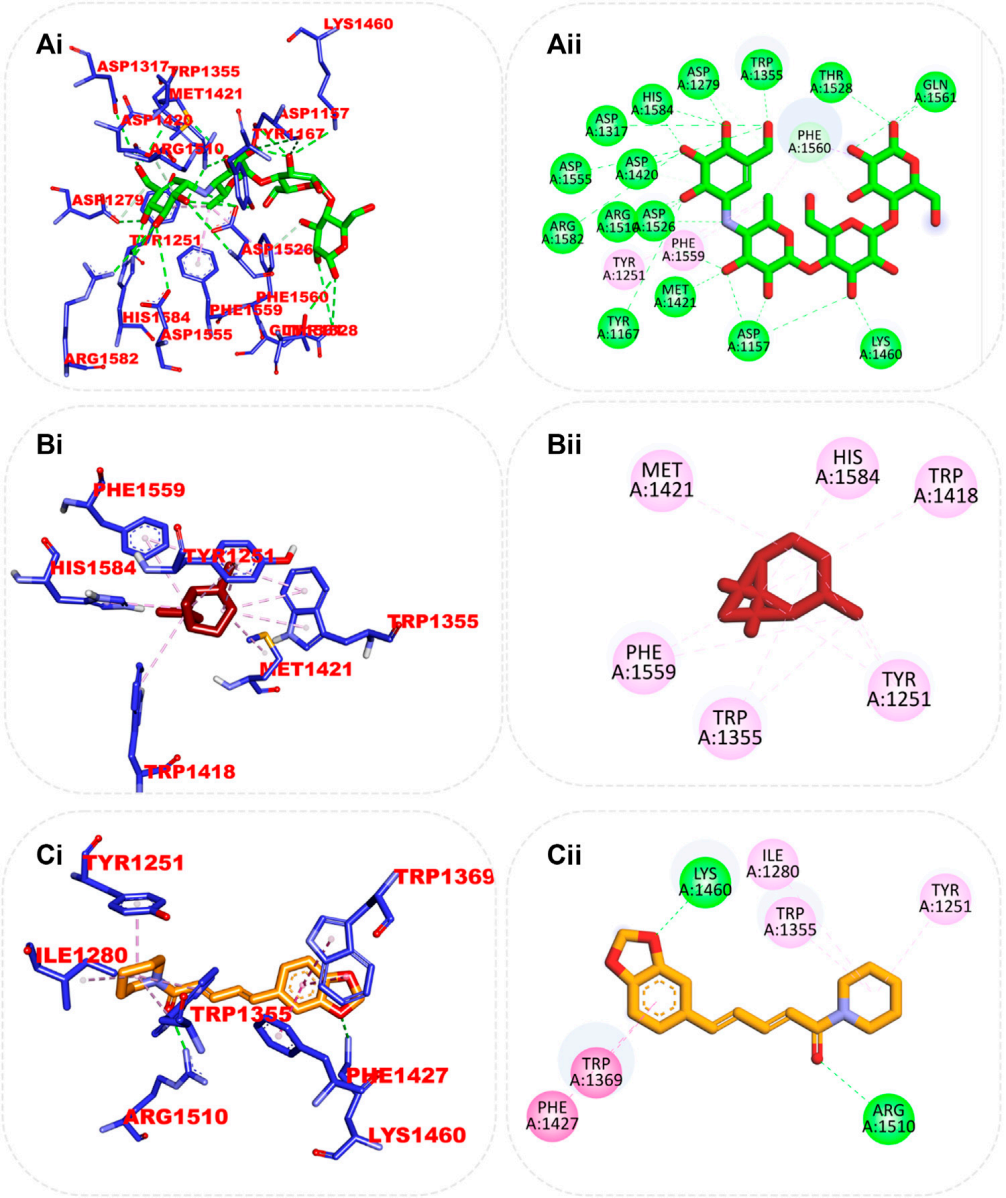
Top ranked secondary metabolites and reference inhibitor (acarbose) from the docking analysis of GCMS phytochemicals from ethyl acetate fraction of *S. filicaulis* leaves in the active site of *human* α-glucosidase. The ligands are displayed as sticks and distinguished by their colors **(Ai)** 3D interaction of acarbose is shown in green, **(Aii)** 2D interaction of acarbose, **(Bi)** 3D interaction of alpha-caryophyllene is presented in red, **(Bii)** 3D interaction of alpha-caryophyllene, and **(Ci)** 3D interaction of piperine is presented in gold, **(Cii)** 2D interaction of piperine.

Although the orientation of acarbose in the binding site of HPA was stretched into the five subsites, the top docked phytocompounds piperine and 4-hydroxy-3-methylacetophenone were docked into the −3 and −1 subsets of the α-amylase. Piperine did not establish hydrogen bonds like the reference compounds, but it did interact with the catalytic residues at the hydrophobic gate of α-amylase, which are Trp-59, Tyr62, and His299. BCQDM made several hydrogen bonds with the catalytic residues, including Asp197, Glu233, Arg195, Ala198, and His305; a pi-sigma contact with Leu165; and carbon hydrogen contacts with Asp197 and Glud233 (Figure 6). The piperine formed one hydrogen bond with Lys1460 and Arg1510, pi-pi stacking, and pi-pi-T-shaped stacking with Phe1427 and Trp1369 of HG. The 1-piperoyl moiety made pi-alkyl contact with Trp1355 and alkyl contact with Ile1280 and Tyr1251. While the bond between alpha-caryophyllene and HG with pi-alkyl contact with Phe1560 and Trp1355 (Figure 7).

#### 3.7.4 Cluster analysis and ensemble-based docking of GCMS-identified phytoconstituents from ethyl acetate fraction of S. filicaulis leaves with conformers of dipeptidyl peptidase IV, α-amylase, and α-glucosidase enzyme

**FIGURE 8:**
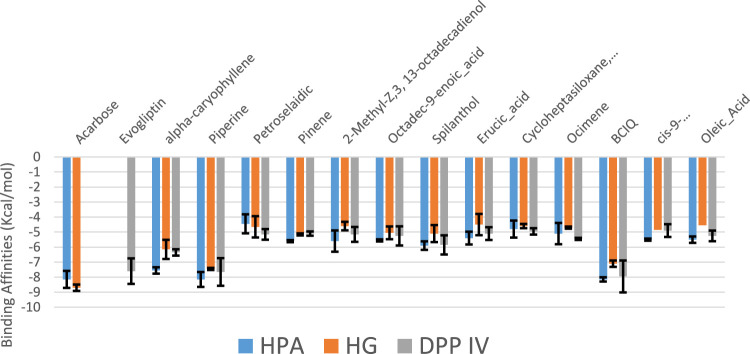
Average binding energies of the acarbose, evogliptin, and GCMS-identified phytoconstituents against representative conformation obtained from the clustering analysis of the MD simulation trajectories of DPP IV, α-amylase and α-glucosidase enzyme. Clusters counts for HPA (2), HG (4) and DPP IV (4)

**FIGURE 9:**
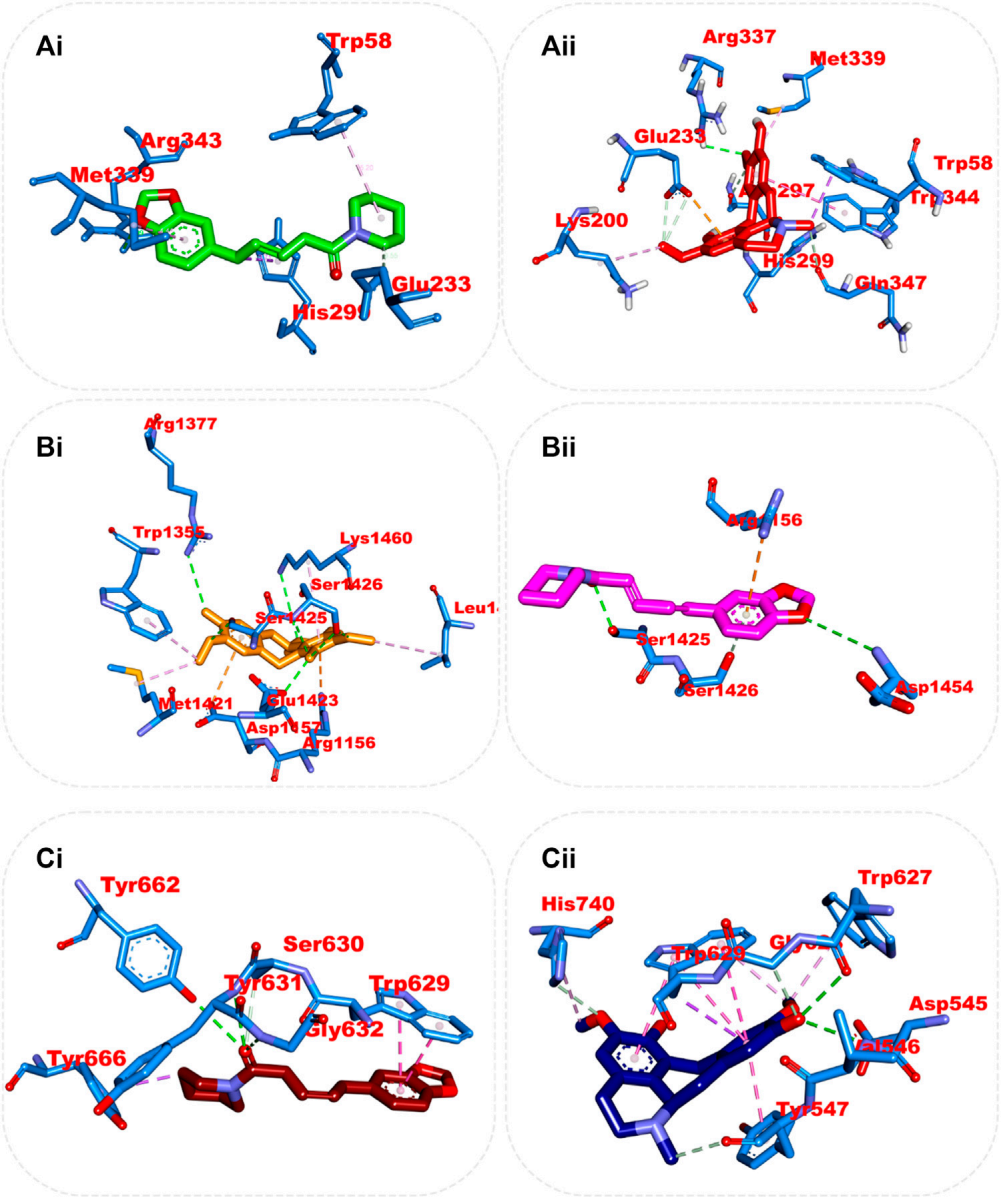
Amino acid interaction of two top docked phytochemicals (piperine and BCQDM) obtained from the ensembled-based docking studies with the representative conformation of **(Ai)** α-amylase with piperine, **(Aii)** α-amylase with BCQDM, **(Bi)** α-glucosidase with piperine, **(Bii)** α-glucosidase with BCQDM, and **(Ci)** DPP IV with the lowest binding affinity with piperine, **(Cii)** DPP IV and BCQDM.

From the trajectories obtained from the MDS analysis of the dipeptidyl peptidase IV, α-amylase, and α-glucosidase enzymes, the RMSD and RMSF plots were calculated to measure the extent of fluctuation during the simulation period (Supplementary Figures S3, S4). We obtained 1000 conformers of the 1000 frames from the MD simulation trajectories using TTclust to provide 4, 2, and 4 clusters for dipeptidyl peptidase, α-amylase, and α-glucosidase, respectively. The Supplementary Material shows the dimensions of the cluster, which are the number of individual frames that make up the clusters, the number of frames of the representative conformation, and the spread, which is the average distance among the conformations that make up the cluster. From these clusters, representative conformations were selected. An ensemble docking was performed by docking the phytochemicals to the representative structures of the various conformers. We calculated the mean and standard deviation for each enzyme of the phytochemical docking scores with minimal energy for each conformation (Figure 8). The results of the ensemble-based docking analysis further confirmed piperine as the phytochemical with the highest binding affinities to DPP IV (−7.65 ± 0.92 Kcal/mol), α-amylase (−8.15 ± 0.50 Kcal/mol), and α-glucosidase (−7.45 ± 0.468 Kcal/mol), while benzocycloheptano[2,3,4-I,j]isoquinoline, 4,5,6,6a-tetrahydro-1,9-dihydroxy-2,10-dimethoxy-5-methyl was the second top docked phytochemical to DPP IV (−7.95 ± 1.06 Kcal/mol), α-amylase (−8.14 ± 0.05 KKcal/mol), and α-glucosidase (−7. 1 ± 0.14 Kcal/mol) respectively. The representative cluster with the lowest binding affinities to the best-docked phytochemicals was selected for interactive analysis and is presented in Figure 9.

#### 3.7.5 Top-docked steroidal saponins’ drug-likeness and pharmacokinetic characteristics

Predictive drug-likeness and ADMET (absorption, distribution, metabolism, excretion, and toxicity) filtering studies were conducted on the two hit compounds that were obtained from the ensemble-based docking analysis. The results are shown in Supplementary Tables S2, S3. The two top-docked phytochemicals, piperine and BCQDM, fulfilled the requirement for the four filters (Lipinki, Veber, Ghose, and Egan), hence they are predicted to have favorable druggable properties.

**FIGURE 10:**
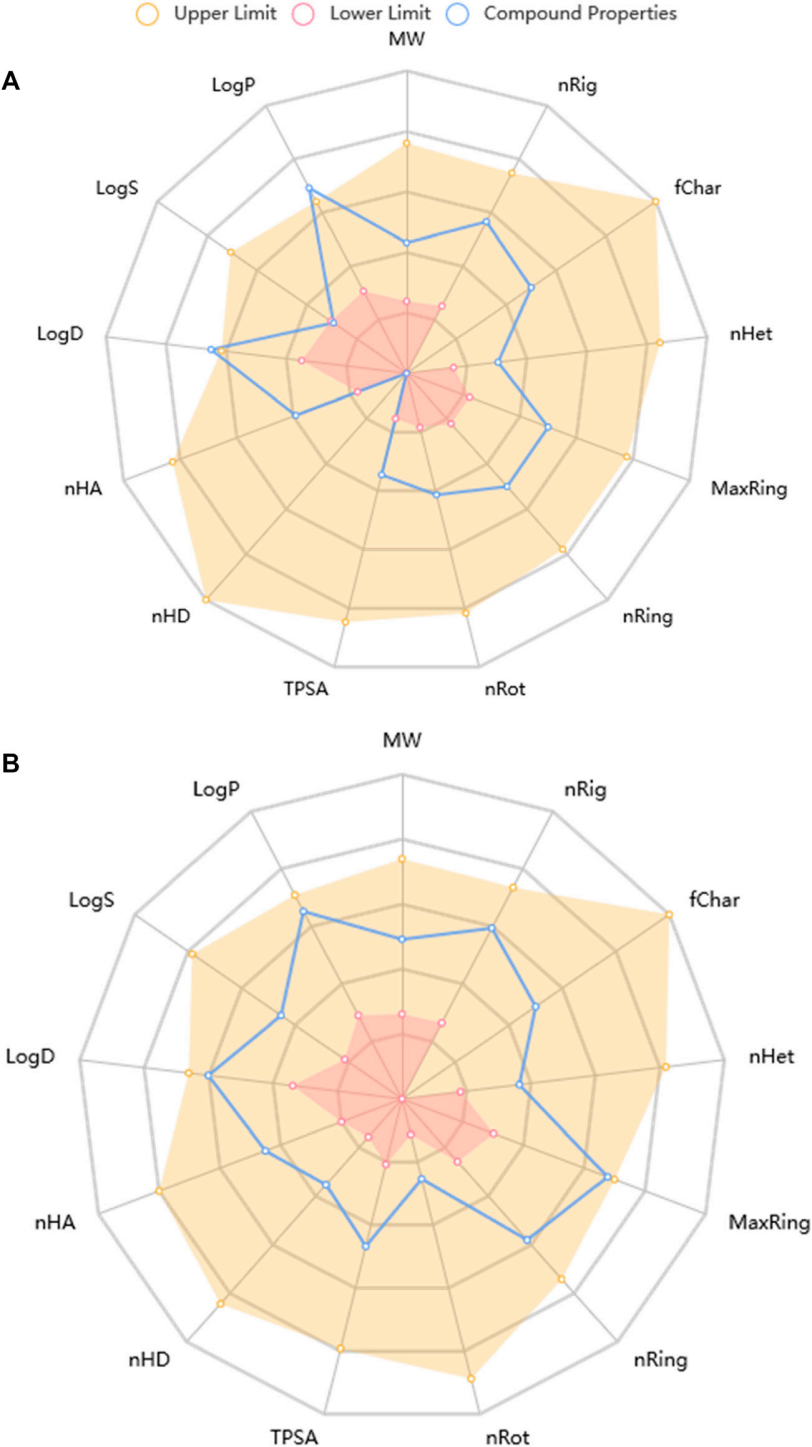
Physicochemical Property of top docked phytochemicals **(A)** Piperine and **(B)** Benzocycloheptano[2,3,4-I,j]isoquinoline, 4,5,6,6a-tetrahydro-1,9-dihydroxy-2,10-dimethoxy-5-methyl.

Piperine and BCQDM were further subjected to predictive ADMET analysis. The substantial gastrointestinal absorption of piperine and BCQDM suggests high bioavailability. Both piperine and BCQDM demonstrated the ability to cross the blood-brain barrier, which is a crucial characteristic of medications used for neurotherapeutic purposes (Supplementary Table S2). Piperine and BCQDM were predicted to be negative substrates of the p-glycoprotein with very high plasma protein binding tendencies. A variety of molecular cytochrome P450 descriptors were employed to investigate the effects of lead metabolites on the biotransformation of drugs in the liver. It was concluded that these descriptors would not be inhibited by piperine and BCQDM. Lead compounds were neither mutagenic, carcinogenic, nor likely to cause skin sensitivity, according to the prediction analysis. The projected LD_50_, half-life, and clearance rate of the lead phytochemicals fell within an acceptable range (Supplementary Table S2; Figure 10).

## 4 Discussion

The use of plant extracts is gaining attention in combating various diseases and ailments, and this is because of the vast phytochemicals present in them that help in mopping up the free radicals present in the systems of the body (Ojo et al., 2020). Oxidative stress arises because unpaired electrons in the system react with proteins and enzymes in a process called glycation (Ahmad et al., 2023). We expect this disease to have a double-digit prevalence in the coming years. It is therefore of great importance that the disease be managed properly to suppress its coming prevalence (Ajiboye et al., 2020). Phytochemicals (phenolics and flavonoids) are known constituents of plant tissues, and they are the main antioxidant driver in plants, which makes them very potent in combating oxidative stress-related diseases. It is no news that year in and year out, researchers have continued to link both phenolics and flavonoids to their antioxidant potential (Kosakowska et al., 2021). We screened the extract of the plant for potential phytochemicals, and we show the resulting outcomes in Table 2.

This research, for the first time, makes use of the leaf part of theS. filicaulis plant in a diabetic study, and according to the result of the in vitroanalysis, DPPH scavenging radicals are well-known for their extraordinary scavenging ability, and from our fraction, it was observed that there was an elevated degree of DPPH quenching potential. This implies that EFSFL may contain beneficial antioxidants capable of removing free radicals from the system. FRAP analysis also signifies the potential of ESSFL to combat ROS, thus reducing the chances of oxidative stress. Our findings support earlier research that suggested plants with reducing and DPPH scavenging abilities do so because they can donate hydrogen to free radicals (Ajiboye et al., 2020; Ojo et al., 2020; Ojo et al., 2022; Ahmad et al., 2023).

In this study, we carried out the inhibition of α-amylase and α-glucosidase by EFSFL. The results analyzed showed that the plant extract expressed the inhibitory activity of these enzymes. α-Amylase reduces the elevated level of glucose in the blood after eating by reducing the rate at which starch is converted to sugar. α-Glucosidase inhibitory potential was also increased in the extract, which shows that the plant has the potential to combat hyperglycemia (Kartini et al., 2023). They prevent the digestion of carbohydrates, and they are competitive inhibitors. Also, prevent the conversion of carbohydrates to glucose. According to the findings of this study, EFSFL was shown to possess chelating radical scavenging activities, such that as the concentration increases, the ion chelating ability also increases and the aqueous extract could chelate Fe^2+^ in a concentration-dependent manner. We could attribute this chelating ability to the existence of phytochemicals with antioxidant properties in the extract. According to (Sarkar et al., 2012; Ojo et al., 2022), iron encourages the process of creating reactive oxygen species (ROS), which can stimulate the peroxidation of lipids. When iron II (Fe^2+^) reacts with H_2_O_2_ through the Fenton reaction, it generates an extremely reactive hydroxyl radical. This radical has detrimental effects on protein, lipid, and nucleic acid processes. As a result, EFSFL Fe2+-chelating activity might be useful in managing or preventing disorders like neurological disorders.

DPP-IV inhibitors are a novel way to manage type 2 diabetes. The pre-meal insulin secretion stimulant glucagon-like peptide-1 (GLP-1) and the glucose-dependent insulinotropic peptide (GIP) are both improved by DPP-IV inhibitors (Borde et al., 2016). DPP-IV inhibition has become an appealing treatment option because various DPP-IV inhibitors consistently lowered blood glucose, primarily postprandially, and this is connected with increases in active circulating glucagon-like peptide-1 (GLP-1) (Green et al., 2006). DPP-IV inhibitors increase GLP-1 and GIP, decreasing glucagon release, which boosts insulin secretion and decreases gastric emptying (Pathak and Brideman, 2010). Several studies have shown the anti-diabetic properties of medicinal plant extracts, but this is the first to indicate that the ethyl acetate fraction of *S. filicaulis* exhibits strong DPP-IV inhibitory activity. EFSFL showed concentration-dependent inhibition (the percentage of inhibition increases as the concentration increases) activity against DPP-IV in our study, with IC_50_ values of 380.94 μg/mL. On the other hand, standard evogliptin exhibited strong action against DPP-IV with IC_50_ values of 211.35 μg/mL. The inhibitory action of EFSFL was comparable to that of the synthetic DPP-IV inhibitor evogliptin. Thus, based on the findings, it is possible to conclude that EFSFL can be an excellent source of indigenously developed DPP-IV inhibitors. The action of EFSFL demonstrated its capacity to prevent incretin from being degraded by DPP-IV into metabolites devoid of insulin-releasing activity.

A molecular docking study ofS. filicaulis leaf extract showed inhibitory activity on DPP-IV. The results of *in silico* analysis showed that there were two top compounds of ethyl acetate fraction of S. filicaulis leaf may serve as DPP inhibitors, that is, alpha-caryophyllene, and piperine. Their binding affinity values were favorable. The top two compounds, alpha-caryophyllene, and piperine, showed a low value in binding free energy. It means that the binding between ligand and molecule target is easy, which causes strong DPP-IV inhibitory activity. The low binding free energy means that these compounds can inhibit DPP-IV activity. We predicted a stronger biological activity for the compound with a higher binding value since it can bind both ligand and molecule targets. This shows comparable interactions and potency with evogliptin.

EFSFL demonstrated that the extract has the capability of stabilizing the membranes of lysosomes and hindering inflammation in tissues (Cigremis et al., 2009). The extract’s ability to inhibit proteinases enhances its ability to inhibit tissue inflammation (Yazdanparast et al., 2008). The increased MDA level in the untreated tissue indicates lipid peroxidation, which is linked to catalase function depletion in the untreated tissue, which means there is a suppression of antioxidant potential. EFSFL treatment improved CAT, SOD activities, and GSH while decreasing MDA levels, indicating a protective effect against oxidative damage produced by ferric-induced oxidation. Previous reports on the use of plants as antioxidants in managing oxidative-related diseases found similar results (Han et al., 2013; Salekzamani et al., 2019; Yue et al., 2020; Tekin and Seven, 2022).

In this research work, the GCMS-identified phytochemicals were docked against DPP IV, HPA, and HG using both molecular docking and ensemble-based docking protocols. The result from the initial docking analysis identified alpha-caryophyllene, piperine, and benzocycloheptano[2,3,4-I,j]isoquinoline, 4,5,6,6a-tetrahydro-1,9-dihydroxy-2,10-dimethoxy-5-methyl as the top docked phytochemicals to the three target proteins. The ensemble-based molecular docking approach in which the phytochemicals were docked to different conformational structures of the targeted proteins that were clustered from the MD simulation trajectory afforded a more in-depth analysis (Amaro et al., 2018) that further confirmed piperine and benzocycloheptano[2,3,4-I,j]isoquinoline, 4,5,6,6a-tetrahydro-1,9-dihydroxy-2,10-dimethoxy-5-methyl as the top docked phytochemicals. We found these compounds to be strongly bound to the catalytic residues of the enzymes. The DPP IV top-docked compounds interacted with residues in the S1 hydrophobic pocket and some residues (Tyr662, Tyr666, Val711, Asn710, Val656, Ser630, and Tpr659) of the pocket and S2 pocket. Among such residues is Phe357 in the S2 extensive subsite, which has been reported to play a vital role in the inhibitory activities of evogliptin (Lee et al., 2017). This interaction corresponds to that of sitagliptin (Kim et al., 2005). Also, Asp197 of HPA has been reported to be mainly responsible for the cleavage of the glycosidic bonds in polysaccharides (Zhang et al., 2009; Taha et al., 2019). reported the role of Asp197 as a catalytic nucleophile in hydrolytic reactions and its interaction with known inhibitors. The results from the predictive physiochemical and drug-likeness analysis over several filtering tools revealed piperine and benzocycloheptano[2,3,4-I,j]isoquinoline, 4,5,6,6a-tetrahydro-1,9-dihydroxy-2,10-dimethoxy-5-methyl have druggable drug properties, while 13-octadecenal did not pass the filtering analysis (Daina et al., 2017). Favorable Veber and Lipinski characteristics suggest good penetration, retention, and bioavailability via the mouth (Lipinski, 2000; Veber et al., 2002). The hERG channel plays a vital role in cardiac cells in that the compounds that block the hERG channel during the repolarization and termination stages of an action potential may be responsible for cardiotoxicity. The main phytochemicals did not show signs of being hERG channel blockers, implying that they may not produce cardiotoxicity via the hERG channel (Raschi et al., 2008; Kratz et al., 2017). Both phytochemicals were not substrates for P-gp. Permeability glycoprotein (P-gp) is expressed in the proximal tubular cells of the kidney, liver cells, intestinal epithelium, and capillary endothelial cells comprising the blood-brain barrier and blood-testis barrier, where it pumps xenobiotics back into the urine-conducting ducts, intestinal lumen, bile ducts, and capillaries, respectively (Lin and Yamazaki, 2003). Using a good deal of cytochrome P_450_ descriptors, we also looked into the influence of lead compounds on phase I drug absorption and utilization. The findings showed that the various cytochrome P_450_ had lower inhibitory potential. Suggesting that they might not significantly affect the absorption and utilization of phase I drugs (Kratz et al., 2017). Both phytochemicals were predicted to be within the classified LD_50_ value (Zhu et al., 2009; Djoumbou Feunang et al., 2016) and did not display mutagenicity or carcinogenicity (Xu et al., 2012).

## 5 Conclusion

This study revealed that EFSFL possesses a significant amount of secondary metabolites, which are likely responsible for its antioxidant, antidiabetic, and anti-inflammatory effects. The bioactive constituents identified through GC-MS were found to have antioxidant, anti-inflammatory, and antidiabetic properties, which further supported the results. The study showed that alpha-caryophyllene, piperine, and benzocycloheptano[2,3,4-I,j]isoquinoline, 4,5,6,6a-tetrahydro-1,9-di hydroxy-2,10-dimethoxy-5-methyl (BCQDM) can inhibit the activities of dipeptidyl peptidase IV, α-amylase, and α-glucosidase. It is recommended that additional investigations of the toxicity effects of EFSFL in nonhuman subjects should be done before their medicinal application.

## Data Availability

Data are available on reasonable request from the corresponding author.
