# Supplementary material for: GC-MS chemical profiling, antioxidant, anti-diabetic, and anti-inflammatory activities of ethyl acetate fraction of Spilanthes filicaulis (Schumach. and Thonn.) C.D. Adams leaves: experimental and computational studies
Source: Front Pharmacol. 2023 Jul 20;14:1235810. doi: 10.3389/fphar.2023.1235810 (PMC10399624; doi:10.3389/fphar.2023.1235810)
Supplement: Supplementary file 1 [file DataSheet1.docx]

**GC-MS chemical profiling, antioxidant, anti-diabetic, and anti-inflammatory activities of Ethyl Acetate Fraction of *Spilanthes filicaulis* (Schumach. & Thonn.) C.D. Adams Leaves: Experimental and Computational Studies**

**Oluwafemi Adeleke Ojo^1*^, Akingbolabo Daniel Ogunlakin^1^, Gideon Ampoma Gyebi^2^, Damilare IyinKristi Ayokunle^3^, Adeshina Isaiah Odugbemi^1^, Dare Ezekiel Babatunde^4^, Omolola Adenike Ajayi-Odoko^5^, Matthew Iyobhebhe^6^ Samson Chukwuemeka Ezea^7^ Christopher Oloruntoba Akintayo^8^ Ademola Ayeleso^1,9^, Adebola Busola Ojo^10^ Omolara Olajumoke Ojo^10^**

^1^ Phytomedicine, Molecular Toxicology, and Computational Biochemistry Research Laboratory (PMTCB-RL), Department of Biochemistry, Bowen University, Iwo, 232101, Nigeria. ADO: akingbolabo.ogunlakin@bowen.edu.ng; AIO: [adeshina.odugbemi@gmail.com](mailto:adeshina.odugbemi@gmail.com); AA: ademola.ayeleso@bowen.edu.ng

^2^ Department of Biochemistry, Bingham University, Karu, Nigeria**,** GAG: gideonagyebi@gmail.com

^3^  Department of Pure and Applied Biology, Bowen University, Iwo, Nigeria, DIA: damilare.ayokunle@bowen.edu.ng

^4^ Department of Anatomy, Bowen University, Iwo, Nigeria; DEB: dare.babatunde@bowen.edu.ng

^5^ Department of Microbiology, Bowen University, Iwo, Nigeria; OA-O: omolola.ajayi@bowen.edu.ng

^6^ Department of Biochemistry, Landmark University, Omu-Aran, Nigeria, MI: iyobhebhematthew@gmail.com

^7^ Department of Pharmacognosy and Environmental Medicine, University of Nigeria, Nsukka, Nigeria, ESC: [samson.ezea@unn.edu.ng](mailto:samson.ezea@unn.edu.ng)

^8^ Department of Physiology, Afe Babalola University, Ado-Ekiti, Nigeria, COA: tobajoe2001@yahoo.com

^9^ Department of Life and Consumer Sciences, School of Agriculture and LifeSciences, University of South Africa,

Florida Park 1709, Roodepoort, South Africa

^10^ Department of Biochemistry, Ekiti State University, Ado-Ekiti, Nigeria, OOO: olajumoke.ojo@eksu.edu.ng

***** Correspondence: oluwafemi.ojo@bowen.edu.ng; Tel.: +2347037824647

**
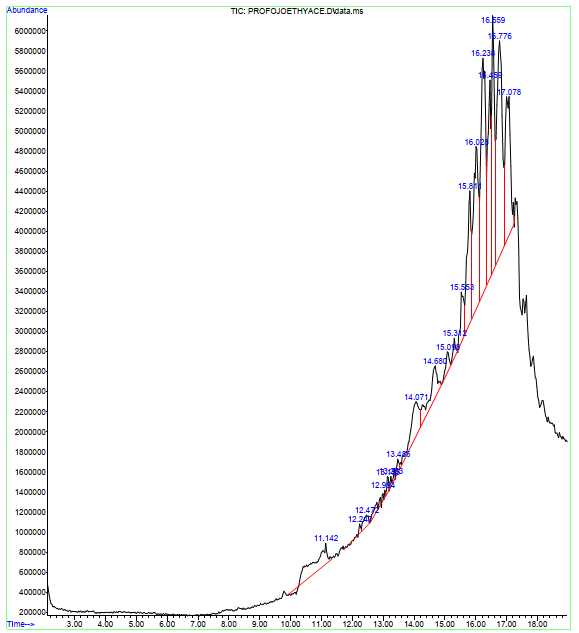
**

**Figure S1.** GC-MS chromatogram of ethyl acetate fraction of *S. filicaulis* leaves


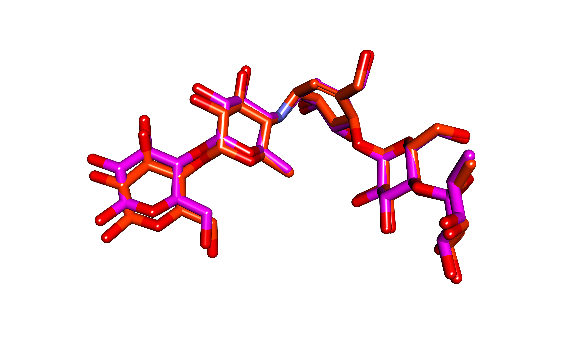


**Figure S2:** Superimposition of docked conformer of the native ligand on the extracted conformation of (a) acarbose. (b) **evogliptin** Red: selected docked conformer and purple: extracted native ligand conformer.

**Figure S3:** The Backbone-RMSD plots of molecular dynamics (MD) simulation of unbound enzymes

**Figure S4**: Per residue RMSF plots of molecular dynamics (MD) simulation of unbound

**Table S1:** Binding site coordinates of the target enzymes

| Dimensions | **DPP IV** | **HPA** (Å) | **HG** (Å) |
| --- | --- | --- | --- |
| center_x | 46.59 | 19.75 | 17.18 |
| center_y | -14.33 | 5.64 | 18.74 |
| center_z | 55.01 | 47.01 | 26.05 |
| Size x | 28.62 | 26.69 | 21.43 |
| Size y | 25.0 | 22.93 | 23.12 |
| Size z | 25.75 | 22.81 | 21.43 |

**Table S2:** Predicted physiochemical and Druglikeness properties of the top two docked phytochemicals

|  | Piperine | BCQDM |
| --- | --- | --- |
| Molecular weight (g/mol) | 285.34 | 341.40 |
| Num. arom. Heavy atoms | 6 | \|  \| 12 \| \| --- \| --- \| |
| Num. heavy atoms | 21 | 25 |
| Num. H-bond acceptors | 4 | 2 |
| Num. rotatable bonds | 3 | 5 |
| Hydrogen bond donor | 0 | 2 |
| MLogP | 2.39 | 1.98 |
| Molar Refractivity | 85.47 | 100.2 |
| TPSA (Å²) | 38.77 | 62.16 |
| **Drug-likeness** |  |  |
| Lipinski | Yes | Yes |
| Veber | Yes | Yes |
| Ghose | Yes | Yes |
| Egan | Yes | Yes |
| Bioavailability Score | 0.55 | 0.55 |

**Legend:** BCQDM: Benzocycloheptano[2,3,4-I,j]isoquinoline,_4,5,6,6a-tetrahydro-1,9-dihydroxy-2,10-dimethoxy-5-methyl

**Table S3:** Predicted ADMET^a^ parameters of the top two docked phytochemicals

|  | **Piperine** | **Benzocycloheptano[2,3,4-I,j]isoquinoline, 4,5,6,6a-tetrahydro-1,9-di hydroxy-2,10-dimethoxy-5-methyl** |
| --- | --- | --- |
| **Absorption (Probability)** |  |  |
| **(b) Admet SAR** |  |  |
| HIA (Human Intestinal Absorption) | HIA+ (1.0) | HIA+ (0.958) |
| Blood-Brain Barrier | BBB+(0.9996) | BBB+(0.951) |
| P-glycoprotein Substrate | Neg. (0.0.474) | Neg. (0.8838) |
| Caco-2 Permeability Cm/s | Caco2+ (0.0.644) | Caco2+ (0.785) |
| **Distribution (Probability)** |  |  |
| PPB (Plasma Protein Binding) % | 89.021 | 77.795 |
| VD L/kg | 0.579 | 1.21 |
| **Metabolism (Probability)** |  |  |
| CYP450 1A2 Inhibitor | Pos. (0.968) | Pos. (0.547) |
| CYP450 1A2 Substrate | Neg. (0.463) | Neg. (0.423) |
| CYP450 3A4 Substrate | Pos. (0.931) | Neg. (0.028) |
| CYP450 3A4 Inhibitor | Pos. (0.53) | Neg. (0.674) |
| CYP4502C9 Inhibitor | Neg. (0.285) | Neg. (0.059) |
| CYP450 2C9 Substrate | Neg. (0.499) | Neg. (0.346) |
| CYP4502C19 Inhibitor | Neg. (0.489) | Neg. (0.037) |
| CYP450 2C19 Substrate | Neg. (0589) | Pos. (0.464) |
| CYP4502D6 Inhibitor | Neg. (0.586) | Pos. (0.503) |
| CYP450 2D6 Substrate | Neg. (0.212) | Neg. (0.651) |
| **Elimination** |  |  |
| T _1/2_ (Half Life Time) | 1.483 h | 1.78 h |
| CL (Clearance Rate) mL/min/kg | 1.965 | 2.231 |
| **Toxicity** |  |  |
| hERG Blockers | Neg. (0.911) | Neg. (0.585) |
| AMES | Neg. (0.903) | Neg. (0.318) |
| Carcinogen | Neg. (0.938) | Neg. (0.779) |
| DILI (Drug Induced Liver Injury) | Neg (0.356) | Neg. (0.304) |
| LD_50_ (LD_50_ of acute toxicity) | 2.385 -log mol/kg (1184.044 mg/kg) | 2.762 -log mol/kg (590.571mg/kg) |

Cluster Results for **dipeptidyl peptidase IV (DPP IV)**

cluster 1

size = 158

representative frame=60

spread : 2.27

Frames : [1, 2, 3, 4, 5, 6, 7, 8, 9, 10, 11, 12, 13, 14, 15, 16, 17, 18, 19, 20, 21, 22, 23, 24, 25, 26, 27, 28, 29, 30, 31, 32, 33, 34, 35, 36, 37, 38, 39, 40, 41, 42, 43, 44, 45, 46, 47, 48, 49, 50, 51, 52, 53, 54, 55, 56, 57, 58, 59, 60, 61, 62, 63, 64, 65, 66, 67, 68, 69, 70, 71, 72, 73, 74, 75, 76, 77, 78, 79, 80, 81, 82, 83, 84, 85, 86, 87, 88, 89, 90, 91, 92, 93, 94, 95, 96, 97, 98, 99, 100, 101, 102, 103, 104, 105, 106, 107, 108, 109, 110, 111, 112, 113, 114, 115, 116, 117, 118, 119, 120, 121, 122, 123, 124, 125, 126, 127, 128, 129, 130, 131, 132, 133, 134, 135, 136, 137, 138, 139, 140, 141, 142, 143, 144, 145, 146, 147, 148, 149, 150, 151, 152, 153, 154, 155, 156, 157, 158]

cluster 2

size = 286

representative frame=332

spread : 2.22

Frames : [159, 160, 161, 162, 163, 164, 165, 166, 167, 168, 169, 170, 171, 172, 173, 174, 175, 176, 177, 178, 179, 180, 181, 182, 183, 184, 185, 186, 187, 188, 189, 190, 191, 192, 193, 194, 195, 196, 197, 198, 199, 200, 201, 202, 203, 204, 205, 206, 207, 208, 209, 210, 211, 212, 213, 214, 215, 216, 217, 218, 219, 220, 221, 222, 223, 224, 225, 226, 227, 228, 229, 230, 231, 232, 233, 234, 235, 236, 237, 238, 239, 240, 241, 242, 243, 244, 245, 246, 247, 248, 249, 250, 251, 252, 253, 254, 255, 256, 257, 258, 259, 260, 261, 262, 263, 264, 265, 266, 267, 268, 269, 270, 271, 272, 273, 274, 275, 276, 277, 278, 279, 280, 281, 282, 283, 284, 285, 286, 287, 288, 289, 290, 291, 292, 293, 294, 295, 296, 297, 298, 299, 300, 301, 302, 303, 304, 305, 306, 307, 308, 309, 310, 311, 312, 313, 314, 315, 316, 317, 318, 319, 320, 321, 322, 323, 324, 325, 326, 327, 328, 329, 330, 331, 332, 333, 334, 335, 336, 337, 338, 339, 340, 341, 342, 343, 344, 345, 346, 347, 348, 349, 350, 351, 352, 353, 354, 355, 356, 357, 358, 359, 360, 361, 362, 363, 364, 365, 366, 367, 368, 369, 370, 371, 372, 373, 374, 375, 376, 377, 378, 379, 380, 381, 382, 383, 384, 385, 386, 387, 388, 389, 390, 391, 392, 393, 394, 395, 396, 397, 398, 399, 400, 401, 402, 403, 404, 405, 406, 407, 408, 409, 410, 411, 412, 413, 414, 415, 416, 417, 418, 419, 420, 421, 423, 424, 425, 426, 427, 428, 429, 430, 431, 432, 433, 434, 435, 436, 437, 438, 439, 440, 441, 442, 443, 444, 448]

cluster 3

size = 302

representative frame=621

spread : 2.14

Frames : [422, 445, 446, 447, 449, 450, 451, 452, 453, 454, 455, 456, 457, 458, 459, 460, 461, 462, 463, 464, 465, 466, 467, 468, 469, 470, 471, 472, 473, 474, 475, 476, 477, 478, 479, 480, 481, 482, 483, 484, 485, 486, 487, 488, 489, 490, 491, 492, 493, 494, 495, 496, 497, 498, 499, 500, 501, 502, 503, 504, 505, 506, 507, 508, 509, 510, 511, 512, 513, 514, 515, 516, 517, 518, 519, 520, 521, 522, 523, 524, 525, 526, 527, 528, 529, 530, 531, 532, 533, 534, 535, 536, 537, 538, 539, 540, 541, 542, 543, 544, 545, 546, 547, 548, 549, 550, 551, 552, 553, 554, 555, 556, 557, 558, 559, 560, 561, 562, 563, 564, 565, 566, 567, 568, 569, 570, 571, 572, 573, 574, 575, 576, 577, 578, 579, 580, 581, 582, 583, 584, 585, 586, 587, 588, 589, 590, 591, 592, 593, 594, 595, 596, 597, 598, 599, 600, 601, 602, 603, 604, 605, 606, 607, 608, 609, 610, 611, 612, 613, 614, 615, 616, 617, 618, 619, 620, 621, 622, 623, 624, 625, 626, 627, 628, 629, 630, 631, 632, 633, 634, 635, 636, 637, 638, 639, 640, 641, 642, 643, 644, 645, 646, 647, 648, 649, 650, 651, 652, 653, 654, 655, 656, 657, 658, 659, 660, 661, 662, 663, 664, 665, 666, 667, 668, 669, 670, 671, 672, 673, 674, 675, 676, 677, 678, 679, 680, 681, 682, 683, 684, 685, 686, 687, 688, 689, 690, 691, 692, 693, 694, 695, 696, 697, 698, 699, 700, 701, 702, 703, 704, 705, 706, 707, 708, 709, 710, 711, 712, 713, 714, 715, 716, 717, 718, 719, 720, 721, 722, 723, 724, 725, 726, 728, 729, 730, 731, 732, 733, 734, 735, 736, 738, 739, 740, 741, 742, 743, 744, 745, 746, 758, 768]

cluster 4

size = 255

representative frame=915

spread : 2.09

Frames : [727, 737, 747, 748, 749, 750, 751, 752, 753, 754, 755, 756, 757, 759, 760, 761, 762, 763, 764, 765, 766, 767, 769, 770, 771, 772, 773, 774, 775, 776, 777, 778, 779, 780, 781, 782, 783, 784, 785, 786, 787, 788, 789, 790, 791, 792, 793, 794, 795, 796, 797, 798, 799, 800, 801, 802, 803, 804, 805, 806, 807, 808, 809, 810, 811, 812, 813, 814, 815, 816, 817, 818, 819, 820, 821, 822, 823, 824, 825, 826, 827, 828, 829, 830, 831, 832, 833, 834, 835, 836, 837, 838, 839, 840, 841, 842, 843, 844, 845, 846, 847, 848, 849, 850, 851, 852, 853, 854, 855, 856, 857, 858, 859, 860, 861, 862, 863, 864, 865, 866, 867, 868, 869, 870, 871, 872, 873, 874, 875, 876, 877, 878, 879, 880, 881, 882, 883, 884, 885, 886, 887, 888, 889, 890, 891, 892, 893, 894, 895, 896, 897, 898, 899, 900, 901, 902, 903, 904, 905, 906, 907, 908, 909, 910, 911, 912, 913, 914, 915, 916, 917, 918, 919, 920, 921, 922, 923, 924, 925, 926, 927, 928, 929, 930, 931, 932, 933, 934, 935, 936, 937, 938, 939, 940, 941, 942, 943, 944, 945, 946, 947, 948, 949, 950, 951, 952, 953, 954, 955, 956, 957, 958, 959, 960, 961, 962, 963, 964, 965, 966, 967, 968, 969, 970, 971, 972, 973, 974, 975, 976, 977, 978, 979, 980, 981, 982, 983, 984, 985, 986, 987, 988, 989, 990, 991, 992, 993, 994, 995, 996, 997, 998, 999, 1000, 1001]

----------------------------

RMSD MATRIX BETWEEN CLUSTERS

+----------+------+------+------+------+

| Clusters | C1 | C2 | C3 | C4 |

+----------+------+------+------+------+

| C1 | 0.00 | 3.03 | 3.46 | 4.69 |

| C2 | 3.03 | 0.00 | 2.43 | 3.67 |

| C3 | 3.46 | 2.43 | 0.00 | 3.03 |

| C4 | 4.69 | 3.67 | 3.03 | 0.00 |

+----------+------+------+------+------+

AVERAGE RSMD BETWEEN CLUSTERS : 3.38


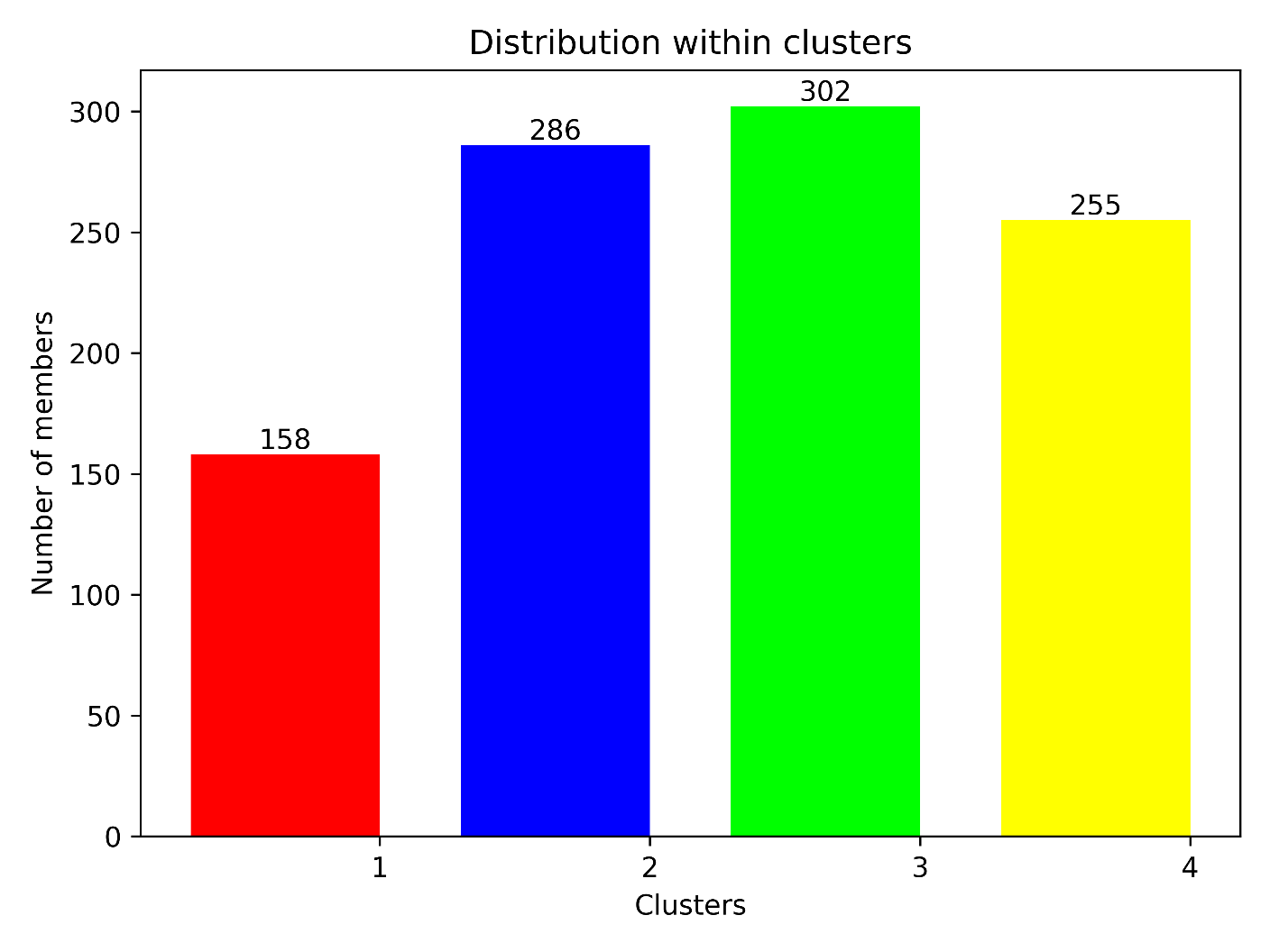


**Cluster Results for α-amylase**

cluster 1

size = 292

representative frame=232

spread : 2.61

Frames : [1, 2, 3, 4, 5, 6, 7, 8, 9, 10, 11, 12, 13, 14, 15, 16, 17, 18, 19, 20, 21, 22, 23, 24, 25, 26, 27, 28, 29, 30, 31, 32, 33, 34, 35, 36, 37, 38, 39, 40, 41, 42, 43, 44, 45, 46, 47, 48, 49, 50, 51, 52, 53, 54, 55, 56, 57, 58, 59, 60, 61, 62, 63, 64, 65, 66, 67, 68, 69, 70, 71, 72, 73, 74, 75, 76, 77, 78, 79, 80, 81, 82, 83, 84, 85, 86, 87, 88, 89, 90, 91, 92, 93, 94, 95, 96, 97, 98, 99, 100, 101, 102, 103, 104, 105, 106, 107, 108, 109, 110, 111, 112, 113, 114, 115, 116, 117, 118, 119, 120, 121, 122, 123, 124, 125, 126, 127, 128, 129, 130, 131, 132, 133, 134, 135, 136, 137, 138, 139, 140, 141, 142, 143, 144, 145, 146, 147, 148, 149, 150, 151, 152, 153, 154, 155, 156, 157, 158, 159, 160, 161, 162, 163, 164, 165, 166, 167, 168, 169, 170, 171, 172, 173, 174, 175, 176, 177, 178, 179, 180, 181, 182, 183, 184, 185, 186, 187, 188, 189, 190, 191, 192, 193, 194, 195, 196, 197, 198, 199, 200, 201, 202, 203, 204, 205, 206, 207, 208, 209, 210, 211, 212, 213, 214, 215, 216, 217, 218, 219, 220, 221, 222, 223, 224, 225, 226, 227, 228, 229, 230, 231, 232, 233, 234, 235, 236, 237, 238, 239, 240, 241, 242, 243, 244, 245, 246, 247, 248, 249, 250, 251, 252, 253, 254, 255, 256, 257, 258, 259, 260, 261, 262, 263, 264, 265, 266, 267, 268, 269, 270, 271, 272, 273, 274, 275, 276, 277, 278, 279, 280, 281, 282, 283, 284, 285, 286, 287, 288, 289, 290, 291, 292]

cluster 2

size = 709

representative frame=677

spread : 2.44

Frames : [293, 294, 295, 296, 297, 298, 299, 300, 301, 302, 303, 304, 305, 306, 307, 308, 309, 310, 311, 312, 313, 314, 315, 316, 317, 318, 319, 320, 321, 322, 323, 324, 325, 326, 327, 328, 329, 330, 331, 332, 333, 334, 335, 336, 337, 338, 339, 340, 341, 342, 343, 344, 345, 346, 347, 348, 349, 350, 351, 352, 353, 354, 355, 356, 357, 358, 359, 360, 361, 362, 363, 364, 365, 366, 367, 368, 369, 370, 371, 372, 373, 374, 375, 376, 377, 378, 379, 380, 381, 382, 383, 384, 385, 386, 387, 388, 389, 390, 391, 392, 393, 394, 395, 396, 397, 398, 399, 400, 401, 402, 403, 404, 405, 406, 407, 408, 409, 410, 411, 412, 413, 414, 415, 416, 417, 418, 419, 420, 421, 422, 423, 424, 425, 426, 427, 428, 429, 430, 431, 432, 433, 434, 435, 436, 437, 438, 439, 440, 441, 442, 443, 444, 445, 446, 447, 448, 449, 450, 451, 452, 453, 454, 455, 456, 457, 458, 459, 460, 461, 462, 463, 464, 465, 466, 467, 468, 469, 470, 471, 472, 473, 474, 475, 476, 477, 478, 479, 480, 481, 482, 483, 484, 485, 486, 487, 488, 489, 490, 491, 492, 493, 494, 495, 496, 497, 498, 499, 500, 501, 502, 503, 504, 505, 506, 507, 508, 509, 510, 511, 512, 513, 514, 515, 516, 517, 518, 519, 520, 521, 522, 523, 524, 525, 526, 527, 528, 529, 530, 531, 532, 533, 534, 535, 536, 537, 538, 539, 540, 541, 542, 543, 544, 545, 546, 547, 548, 549, 550, 551, 552, 553, 554, 555, 556, 557, 558, 559, 560, 561, 562, 563, 564, 565, 566, 567, 568, 569, 570, 571, 572, 573, 574, 575, 576, 577, 578, 579, 580, 581, 582, 583, 584, 585, 586, 587, 588, 589, 590, 591, 592, 593, 594, 595, 596, 597, 598, 599, 600, 601, 602, 603, 604, 605, 606, 607, 608, 609, 610, 611, 612, 613, 614, 615, 616, 617, 618, 619, 620, 621, 622, 623, 624, 625, 626, 627, 628, 629, 630, 631, 632, 633, 634, 635, 636, 637, 638, 639, 640, 641, 642, 643, 644, 645, 646, 647, 648, 649, 650, 651, 652, 653, 654, 655, 656, 657, 658, 659, 660, 661, 662, 663, 664, 665, 666, 667, 668, 669, 670, 671, 672, 673, 674, 675, 676, 677, 678, 679, 680, 681, 682, 683, 684, 685, 686, 687, 688, 689, 690, 691, 692, 693, 694, 695, 696, 697, 698, 699, 700, 701, 702, 703, 704, 705, 706, 707, 708, 709, 710, 711, 712, 713, 714, 715, 716, 717, 718, 719, 720, 721, 722, 723, 724, 725, 726, 727, 728, 729, 730, 731, 732, 733, 734, 735, 736, 737, 738, 739, 740, 741, 742, 743, 744, 745, 746, 747, 748, 749, 750, 751, 752, 753, 754, 755, 756, 757, 758, 759, 760, 761, 762, 763, 764, 765, 766, 767, 768, 769, 770, 771, 772, 773, 774, 775, 776, 777, 778, 779, 780, 781, 782, 783, 784, 785, 786, 787, 788, 789, 790, 791, 792, 793, 794, 795, 796, 797, 798, 799, 800, 801, 802, 803, 804, 805, 806, 807, 808, 809, 810, 811, 812, 813, 814, 815, 816, 817, 818, 819, 820, 821, 822, 823, 824, 825, 826, 827, 828, 829, 830, 831, 832, 833, 834, 835, 836, 837, 838, 839, 840, 841, 842, 843, 844, 845, 846, 847, 848, 849, 850, 851, 852, 853, 854, 855, 856, 857, 858, 859, 860, 861, 862, 863, 864, 865, 866, 867, 868, 869, 870, 871, 872, 873, 874, 875, 876, 877, 878, 879, 880, 881, 882, 883, 884, 885, 886, 887, 888, 889, 890, 891, 892, 893, 894, 895, 896, 897, 898, 899, 900, 901, 902, 903, 904, 905, 906, 907, 908, 909, 910, 911, 912, 913, 914, 915, 916, 917, 918, 919, 920, 921, 922, 923, 924, 925, 926, 927, 928, 929, 930, 931, 932, 933, 934, 935, 936, 937, 938, 939, 940, 941, 942, 943, 944, 945, 946, 947, 948, 949, 950, 951, 952, 953, 954, 955, 956, 957, 958, 959, 960, 961, 962, 963, 964, 965, 966, 967, 968, 969, 970, 971, 972, 973, 974, 975, 976, 977, 978, 979, 980, 981, 982, 983, 984, 985, 986, 987, 988, 989, 990, 991, 992, 993, 994, 995, 996, 997, 998, 999, 1000, 1001]

----------------------------

RMSD MATRIX BETWEEN CLUSTERS

+----------+------+------+

| Clusters | C1 | C2 |

+----------+------+------+

| C1 | 0.00 | 3.95 |

| C2 | 3.95 | 0.00 |

+----------+------+------+

AVERAGE RSMD BETWEEN CLUSTERS : 3.95


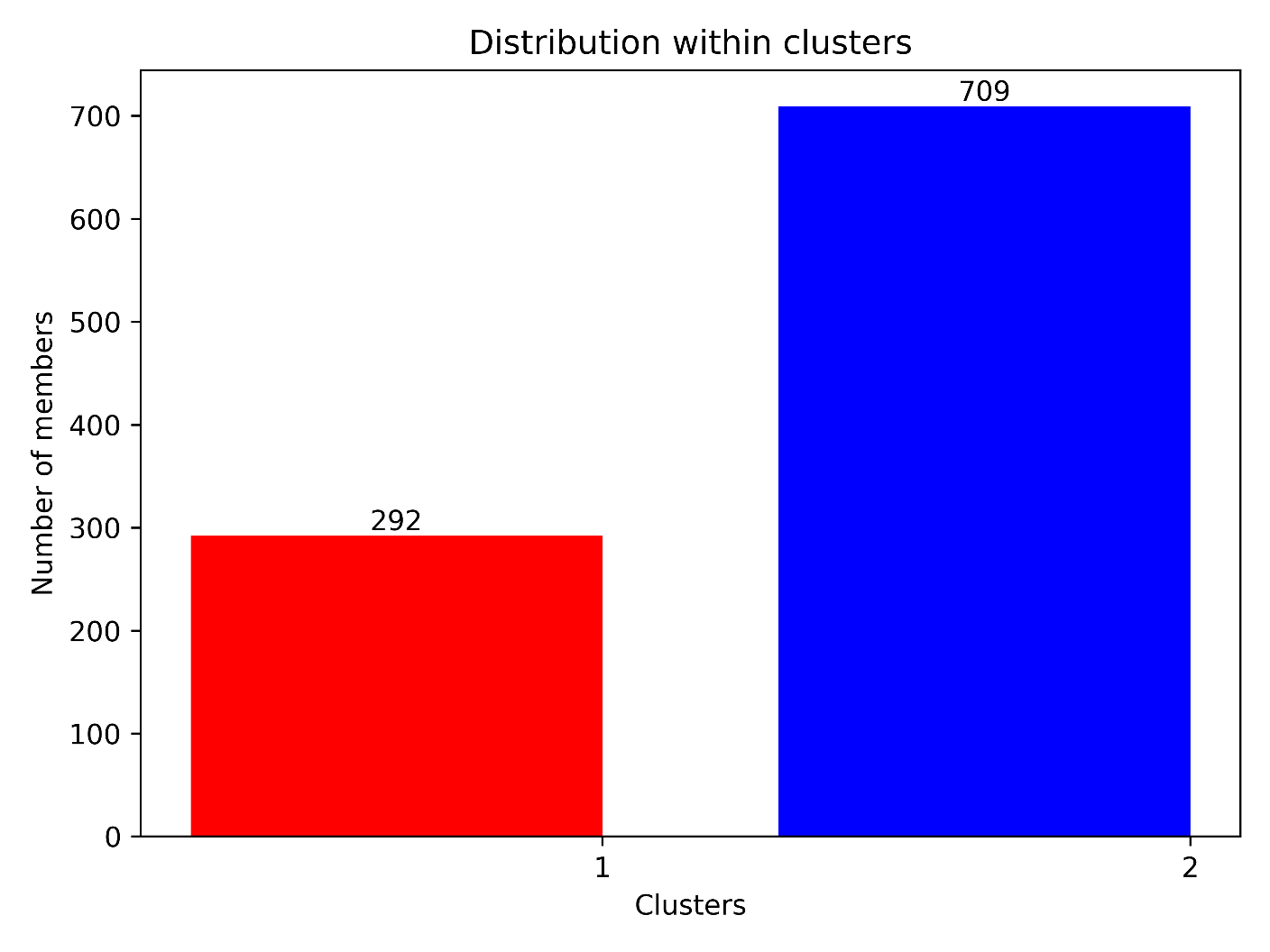


**Cluster Results for α-glucosidase**

cluster 1

size = 263

representative frame=168

spread : 2.35

Frames : [1, 2, 3, 4, 5, 6, 7, 8, 9, 10, 11, 12, 13, 14, 15, 16, 17, 18, 19, 20, 21, 22, 23, 24, 25, 26, 27, 28, 29, 30, 31, 32, 33, 34, 35, 36, 37, 38, 39, 40, 41, 42, 43, 44, 45, 46, 47, 48, 49, 50, 51, 52, 53, 54, 55, 56, 57, 58, 59, 60, 61, 62, 63, 64, 65, 66, 67, 68, 69, 70, 71, 72, 73, 74, 75, 76, 77, 78, 79, 80, 81, 82, 83, 84, 85, 86, 87, 88, 89, 90, 91, 92, 93, 94, 95, 96, 97, 98, 99, 100, 101, 102, 103, 104, 105, 106, 107, 108, 109, 110, 111, 112, 113, 114, 115, 116, 117, 118, 119, 120, 121, 122, 123, 124, 125, 126, 127, 128, 129, 130, 131, 132, 133, 134, 135, 136, 137, 138, 139, 140, 141, 142, 143, 144, 145, 146, 147, 148, 149, 150, 151, 152, 153, 154, 155, 156, 157, 158, 159, 160, 161, 162, 163, 164, 165, 166, 167, 168, 169, 170, 171, 172, 173, 174, 175, 176, 177, 178, 179, 180, 181, 182, 183, 184, 185, 186, 187, 188, 189, 190, 191, 192, 193, 194, 195, 196, 197, 198, 199, 200, 201, 202, 203, 204, 205, 206, 207, 208, 209, 210, 211, 212, 213, 214, 215, 216, 217, 218, 219, 220, 221, 222, 223, 224, 225, 226, 227, 228, 229, 230, 231, 232, 233, 234, 235, 236, 237, 238, 239, 240, 241, 242, 243, 244, 245, 246, 247, 248, 249, 250, 251, 252, 253, 254, 255, 256, 257, 258, 259, 260, 261, 262, 263]

cluster 2

size = 188

representative frame=337

spread : 2.01

Frames : [264, 265, 266, 267, 268, 269, 270, 271, 272, 273, 274, 275, 276, 277, 278, 279, 280, 281, 282, 283, 284, 285, 286, 287, 288, 289, 290, 291, 292, 293, 294, 295, 296, 297, 298, 299, 300, 301, 302, 303, 304, 305, 306, 307, 308, 309, 310, 311, 312, 313, 314, 315, 316, 317, 318, 319, 320, 321, 322, 323, 324, 325, 326, 327, 328, 329, 330, 331, 332, 333, 334, 335, 336, 337, 338, 339, 340, 341, 342, 343, 344, 345, 346, 347, 348, 349, 350, 351, 352, 353, 354, 355, 356, 357, 358, 359, 360, 361, 362, 363, 364, 365, 366, 367, 368, 369, 370, 371, 372, 373, 374, 375, 376, 377, 378, 379, 380, 381, 382, 383, 384, 385, 386, 387, 388, 389, 390, 391, 392, 393, 394, 395, 396, 397, 398, 399, 400, 401, 402, 403, 404, 405, 406, 407, 408, 409, 410, 411, 412, 413, 414, 415, 416, 417, 418, 419, 420, 421, 422, 423, 424, 425, 426, 427, 428, 429, 430, 431, 432, 433, 434, 435, 436, 437, 438, 439, 440, 441, 442, 443, 444, 445, 446, 447, 448, 449, 450, 451]

cluster 3

size = 139

representative frame=501

spread : 1.80

Frames : [452, 453, 454, 455, 456, 457, 458, 459, 460, 461, 462, 463, 464, 465, 466, 467, 468, 469, 470, 471, 472, 473, 474, 475, 476, 477, 478, 479, 480, 481, 482, 483, 484, 485, 486, 487, 488, 489, 490, 491, 492, 493, 494, 495, 496, 497, 498, 499, 500, 501, 502, 503, 504, 505, 506, 507, 508, 509, 510, 511, 512, 513, 514, 515, 516, 517, 518, 519, 520, 521, 522, 523, 524, 525, 526, 527, 528, 529, 530, 531, 532, 533, 534, 535, 536, 537, 538, 539, 540, 541, 542, 543, 544, 545, 546, 547, 548, 549, 550, 551, 552, 553, 554, 555, 556, 557, 558, 559, 560, 561, 562, 563, 564, 565, 566, 567, 568, 569, 570, 571, 572, 573, 574, 575, 576, 577, 578, 579, 580, 581, 582, 583, 584, 585, 586, 587, 588, 589, 590]

cluster 4

size = 411

representative frame=766

spread : 1.98

Frames : [591, 592, 593, 594, 595, 596, 597, 598, 599, 600, 601, 602, 603, 604, 605, 606, 607, 608, 609, 610, 611, 612, 613, 614, 615, 616, 617, 618, 619, 620, 621, 622, 623, 624, 625, 626, 627, 628, 629, 630, 631, 632, 633, 634, 635, 636, 637, 638, 639, 640, 641, 642, 643, 644, 645, 646, 647, 648, 649, 650, 651, 652, 653, 654, 655, 656, 657, 658, 659, 660, 661, 662, 663, 664, 665, 666, 667, 668, 669, 670, 671, 672, 673, 674, 675, 676, 677, 678, 679, 680, 681, 682, 683, 684, 685, 686, 687, 688, 689, 690, 691, 692, 693, 694, 695, 696, 697, 698, 699, 700, 701, 702, 703, 704, 705, 706, 707, 708, 709, 710, 711, 712, 713, 714, 715, 716, 717, 718, 719, 720, 721, 722, 723, 724, 725, 726, 727, 728, 729, 730, 731, 732, 733, 734, 735, 736, 737, 738, 739, 740, 741, 742, 743, 744, 745, 746, 747, 748, 749, 750, 751, 752, 753, 754, 755, 756, 757, 758, 759, 760, 761, 762, 763, 764, 765, 766, 767, 768, 769, 770, 771, 772, 773, 774, 775, 776, 777, 778, 779, 780, 781, 782, 783, 784, 785, 786, 787, 788, 789, 790, 791, 792, 793, 794, 795, 796, 797, 798, 799, 800, 801, 802, 803, 804, 805, 806, 807, 808, 809, 810, 811, 812, 813, 814, 815, 816, 817, 818, 819, 820, 821, 822, 823, 824, 825, 826, 827, 828, 829, 830, 831, 832, 833, 834, 835, 836, 837, 838, 839, 840, 841, 842, 843, 844, 845, 846, 847, 848, 849, 850, 851, 852, 853, 854, 855, 856, 857, 858, 859, 860, 861, 862, 863, 864, 865, 866, 867, 868, 869, 870, 871, 872, 873, 874, 875, 876, 877, 878, 879, 880, 881, 882, 883, 884, 885, 886, 887, 888, 889, 890, 891, 892, 893, 894, 895, 896, 897, 898, 899, 900, 901, 902, 903, 904, 905, 906, 907, 908, 909, 910, 911, 912, 913, 914, 915, 916, 917, 918, 919, 920, 921, 922, 923, 924, 925, 926, 927, 928, 929, 930, 931, 932, 933, 934, 935, 936, 937, 938, 939, 940, 941, 942, 943, 944, 945, 946, 947, 948, 949, 950, 951, 952, 953, 954, 955, 956, 957, 958, 959, 960, 961, 962, 963, 964, 965, 966, 967, 968, 969, 970, 971, 972, 973, 974, 975, 976, 977, 978, 979, 980, 981, 982, 983, 984, 985, 986, 987, 988, 989, 990, 991, 992, 993, 994, 995, 996, 997, 998, 999, 1000, 1001]

----------------------------

RMSD MATRIX BETWEEN CLUSTERS

+----------+------+------+------+------+

| Clusters | C1 | C2 | C3 | C4 |

+----------+------+------+------+------+

| C1 | 0.00 | 3.17 | 3.54 | 3.74 |

| C2 | 3.17 | 0.00 | 2.68 | 2.84 |

| C3 | 3.54 | 2.68 | 0.00 | 2.54 |

| C4 | 3.74 | 2.84 | 2.54 | 0.00 |

+----------+------+------+------+------+

AVERAGE RSMD BETWEEN CLUSTERS : 3.09
